# Methods of assessing value for money of UK-based early childhood public health interventions: a systematic literature review

**DOI:** 10.1093/bmb/ldac035

**Published:** 2022-12-19

**Authors:** Peter Murphy, Sebastian Hinde, Helen Fulbright, Louise Padgett, Gerry Richardson

**Affiliations:** Centre for Health Economics, University of York, York, YO10 5DD, UK; Centre for Health Economics, University of York, York, YO10 5DD, UK; Centre for Reviews and Dissemination, University of York, York, YO10 5DD, UK; Department of Health Sciences, University of York, York, YO10 5DD, UK; Centre for Health Economics, University of York, York, YO10 5DD, UK

**Keywords:** economic evaluation, infant, child, public health

## Abstract

**Introduction:**

Economic evaluation has an important role to play in the demonstration of value for money of early childhood public health interventions; however, concerns have been raised regarding their consistent application and relevance to commissioners. This systematic review of the literature therefore aims to collate the breadth of the existing economic evaluation evidence of these interventions and to identify the approaches adopted in the assessment of value.

**Source of data:**

Recently published literature in Medline, EMBASE, EconLit, Health Management Information Consortium, Cochrane CENTRAL, Cochrane Database of Systematic Reviews, Health Technology Assessment, NHS EED and Web of Science.

**Areas of agreement:**

The importance of the early childhood period on future health and well-being as well as the potential to impact health inequalities making for a strong narrative case for expenditure in early childhood public health.

**Areas of controversy:**

The most appropriate approaches to evaluating value for money of such preventative interventions relevant for UK decision-makers given the evident challenges.

**Growing points:**

The presented review considered inconsistencies across methodological approaches used to demonstrate value for money. The results showed a mixed picture in terms of demonstrating value for money.

**Areas timely for developing research:**

Future resource allocations decisions regarding early childhood public health interventions may benefit from consistency in the evaluative frameworks and health outcomes captured, as well as consistency in approaches to incorporating non-health costs and outcomes, incorporating equity concerns and the use of appropriate time horizons.

## Introduction

The importance of the early childhood period on future health and well-being is well established.^1^^,^^2^ Interventions in early life not only have the potential to impact diseases in adult life but also to impact health inequalities experienced throughout the life course.^3–7^ Policy recommendations in the UK and elsewhere have therefore reiterated the importance of increasing public or government expenditure by local and national decision-makers on those in early childhood^6^^,^^8^ as well as more broadly acknowledging the value of investing in preventative interventions.^9^

Despite the presence of a strong narrative case for prevention in early childhood, it is important that decisions to fund such interventions are based on systematic and robust assessments of clinical and economic evidence. Resources are limited and decisions to fund an intervention means the opportunity to fund alternative interventions are foregone. Economic evaluation provides a systematic and transparent framework to identify which interventions offer value for money and help inform the choice of competing claims on limited resources.^10^

In the UK, methods of health economic evaluation are generally well established when informing health technology assessment decisions.^11^ That is, interventions for treating existing conditions. Yet, when it comes to preventative interventions at a population level, there remains less agreement on the most appropriate methods for conducting economic evaluation.^12^^,^^13^ This is particularly true for public health interventions targeting early childhood given the complex interplay between health, development, education, socioeconomic status and the family environment.^14–16^

The range of methodological challenges of conducting economic evaluations of such early childhood interventions has been highlighted in the literature and includes: appropriate time horizons, measuring and valuing health outcomes, incorporating non-health costs and outcomes, and informing health equity concerns.^17–19^ These challenges may in-part explain why a number of large-scale early childhood preventative interventions have failed to show cost-effectiveness^13^ and why gaps exist between the evidence base and decision-making.^20^

Previous literature reviews in a paediatric setting have focussed on economic evaluations of specific intervention categories, such as vaccinations,^21^ parenting interventions,^22^^,^^23^ health promotion,^24–26^ and oral health.^27^ Furthermore, these reviews have not limited the results to a specific country or jurisdiction. We consider it important to identify the economic evidence relevant to UK public health decision-makers given the positive and normative reasons why results of economic evaluations may differ across jurisdictions.^28^ A literature review of public health intervention decisions made by NICE^29^ focussed on those conducted in a UK context yet there was no limitation by age category and the review focussed on NICE guidance and not the wider evidence base.

This systematic review of the literature therefore aims to achieve a number of goals. First, to collate the breadth of the existing economic evaluation evidence of early childhood public health interventions conducted in a UK context. Second, to describe the methods and approaches adopted in the evidence base to highlight consistencies in the demonstration of value for money. Finally, to critically appraise the quality of the evidence base. By doing so, this review seeks to provide researchers and policymakers in the UK details of the relevant economic evaluation evidence, as well as highlighting the methodological challenges and deficiencies in conducting such analyses.

## Methods

The protocol was registered with PROSPERO (CRD42021270751) and was conducted and reported in accordance with PRISMA guidelines.^30^

### Data sources and searches

An initial search strategy was designed in Ovid MEDLINE with the final strategy adapted with relevant subject headings (controlled vocabularies) and search syntax to each of the databases listed below. No language limits were applied, but papers were limited to 2000 onwards to ensure the relevance to the current research and policy deliberations. Details of the full search strategies are contained in Supplementary Material, Supplementary Appendix 1.

The following databases were searched between August 16 and 23, 2021:

1. MEDLINE(R) ALL (Ovid): 1946 to August 13, 2021.

2. Embase (Ovid): 1974 to August 17, 2021.

3. Econlit (Ovid): 1886 to August 5, 2021.

4. Health Management Information Consortium (HMIC) (Ovid): 1979 to July 2021.

5. Cochrane Central Register of Controlled Trials (Wiley): 2021, Issue 8 in the Cochrane Library.

6. Cochrane Database of Systematic Reviews (Wiley): 2021, Issue 8 in the Cochrane Library.

7. Health Technology Assessment (CRD): Incep-tion to March 2018.

8. Economic Evaluations Database (CRD): Inception to March 31, 2015;

9. Science Citation Index Expanded (Web of Science): 1900 to August 16, 2021.

Supplementary searches in the form of reference checking and backwards citation searching^31^ of systematic reviews identified in the primary database search were undertaken on October 15, 2021.

### Study selection

Two review authors (P.M. and L.P.) independently conducted title and abstract screening of a random sample of 10% of the retrieved records. A kappa statistic for assessing inter-rater agreement^32^ was calculated. Upon the achievement of a kappa statistic of 0.8 or above, one reviewer screened the remaining titles and abstracts. Failure to achieve the required kappa statistic meant a further 10% would be screened by both reviewers until the required score was achieved. Reviewers screened 20% (two screening rounds) before the sufficient kappa statistic was achieved. This process was applied at both the title and abstract screening stage and the full text screening stage. Any discrepancies were resolved by discussion between the two review authors.

Records were included if they reported economic evaluations of public health interventions in the UK. Public health was defined using terms that broadly reflected interventions of health improvement and included terms for wider social determinants of health (Supplementary Material, Supplementary Appendix 1). Evaluations were limited to those of interventions for infants and children with a mean age of 5 years or under at baseline to reflect the infant, toddler and preschool years. Economic evaluations of interventions for ages above 5 years of age were considered for inclusion if they explicitly included a subgroup analysis for those 5 years or under. Those evaluating interventions aimed at pregnant women or for the treatment of existing conditions in infants, children and family members were excluded.

All studies that aimed to inform a value for money assessment of both the cost- and health-related outcomes of an intervention were included. However, the methodological approach taken in the study was stratified into a number of groups conditional on whether they combined the costs and outcomes into a single framework and/or incorporated a comparator intervention.

Cost-effectiveness analyses (CEA) were defined as studies that captured the costs and health outcomes of competing interventions, with health outcomes expressed as either a generic measure of health such as quality-adjusted life years (QALYs), referred to hereafter as ‘QALY-based CEAs’, or expressed in alternative natural units, referred to hereafter as ‘non-QALY-based CEAs’.

Cost-consequence analyses (CCA) were defined as those reporting disaggregated costs and health outcomes of competing interventions; and cost-benefit analysis (CBA) that captures the costs and outcomes, both expressed in monetary terms.

Evaluation frameworks in the form of ‘performance measures’ that consider both costs and outcomes but do not necessarily require a comparative analysis were also included in this review, owing to their inclusion in Public Health England’s Health Economic Evidence Resource (HEER) tool.^33^ This includes social return on investment (SROI) and return on investment (ROI). Both present a ratio of the monetary returns to the money spent, with the former focussing on the wider costs and benefits to society beyond just healthcare.^13^

Sources of grey literature amongst the search results were included in Ovid’s HMIC database, which includes literature on health management, health service policies, public health and social care with an emphasis on the UK and the NHS. Grey literature that was found through supplementary searches of previous systematic reviews was deemed eligible for inclusion.

### Data extraction and critical appraisal

A *de novo* data extraction pro forma was used. The extracted information was based on central characteristics of the included studies: intervention, comparator and the population. The type of evaluation framework used was extracted as well as the perspective adopted, and the associated extent of the costs and outcomes (health and non-health) included in the evaluation. The time horizon, use of decision modelling and information on the type of decision model (such as decision tree or Markov model) were also extracted. Finally, the incorporation of any equity considerations in the economic evaluation and the empirical results of the evaluation were extracted. The pro forma can be found in Supplementary Material, Supplementary Appendix 2.

Critical appraisal of the included studies was conducted through the use of the CHEERS checklist,^10^ which can be found in Supplementary Material Supplementary Appendix 3.

## Results

### Review profile

**Fig. 1:**
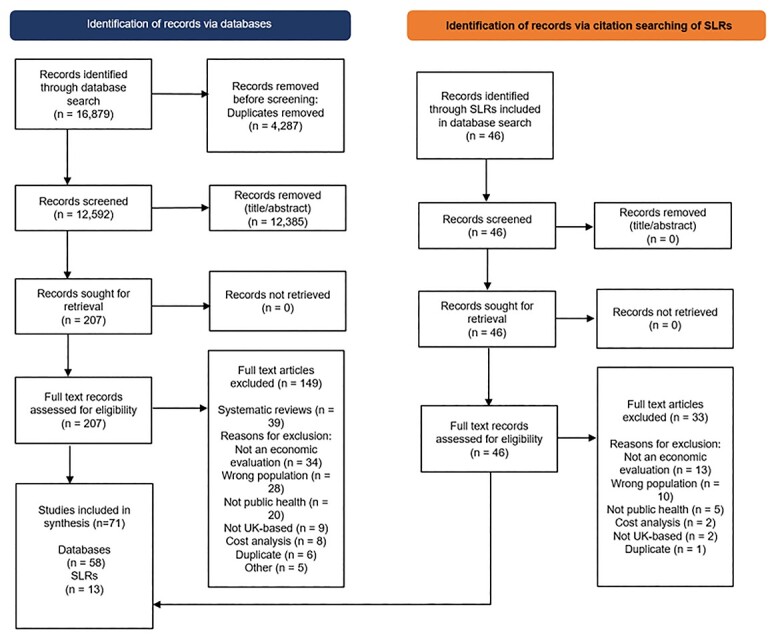
PRISMA diagram of the flow of included and excluded studies.

The database search retrieved 16 879 records resulting in 12 592 unique records following deduplication. Of these, 207 full text articles were screened and 58 met the eligibility criteria. Incitation searching of the previous systematic reviews yielded an additional 13 that met the eligibility criteria. In total, 71 articles were included in the synthesis. See Fig. 1 for the PRISMA flow diagram.^34^ In the case of three papers, the same evaluation was described in two separate papers: Morrell^35^^, 36^, Pandor^36^^,^^37^, and Jacklin^38^ and NICE.^39^ One paper by Kendrick^41^ included two evaluations of differing approaches and interventions. The results of this systematic review are therefore based on 69 individual evaluations.

### Description of results

Health protection programmes were the most common intervention category (32%; 22/69). The remaining interventions were newborn screening (16%; 11/69), parenting support (12%; 8/69), injury prevention (7%; 5/69), health promotion (4%; 3/69), oral health (9%; 6/69), childhood screening (9%; 6/69), breast feeding (6%; 4/69), reducing the risk of maltreatment (3%; 2/69) and finally, interventions that cover both parenting support and health promotion (3%; 2/69). The general characteristics of the identified evaluations can be seen in Table 1.

**Table 1:** Summary of the evaluations

Author year	Intervention category	Intervention (comparator)	Population	Type of evaluation	Study type	Time horizon	Outcomes captured (quality of life instrument)	Primary result of the evaluation
Anokye 2020^40^	Breast feeding	Nourishing Start for Health, NOSH (usual care)	Newborn	Non-QALY-based CEA	Trial based	1 year	Proportion baby breast fed at 6 weeks	£974 per additional breast-fed baby
Hoddinott 2012^41^	Breast feeding	FEeding Support Team, FEST (reactive telephone support)	Newborn	Non-QALY-based CEA	Trial based	6–8 weeks	Any breastfeeding; exclusive breastfeeding	£87 per additional woman any breastfeeding; £91 per additional woman exclusively breast feeding
Jacklin 2007^38^ NICE 2008^39^	Breast feeding	Breast feeding peer support (unclear)	Newborn	QALY-based CEA	Model based	Unclear	QALYs (unclear); premenopausal breast cancer averted; infant infections averted	No ICER reported
Pokhrel 2015^42^	Breast feeding	Breast feeding support (no breast feeding support)	Newborn	CCA (not specified but reports costs and outcomes separately)	Model based	1 year for three acute conditions (GI, LRTI and AOM); lifetime for maternal BC; neonatal unit stay for NEC	Cost savings. Includes a cost derived using NMB assuming 20 000/QALYs for the breast cancer benefits.	Report outcomes using three different types of policies: Policies A, B and C (impacts on acute diseases (GI, LRTI and AOM)); Policy D (impacts NEC) and Policy E (impacts BC). Policy A2 saves £11.04 m; policy D2 saves £6.12 m and policy E2 saves £31.42 m (this includes QALYs gained)
Bamford 2007^43^	Childhood screening	Alternative SES programmes (no SES)	4–5 years	QALY-based CEA	Model based	11 years	QALYs (HUI)	£2445 per QALY
Carlton 2008^44^	Childhood screening	Amblyopia (and strabismus) screening (no screening)	3–5 years	QALY-based CEA	Model based	100 years	QALYs (utility values from the literature)	Screening at 3 years without autorefraction was the most cost-effective, ICER of £527 375 per QALY.
Craig 2011^45^	Childhood screening	Grote strategy for short stature screening (UK strategy)	Under 3 years	QALY-based CEA	Model based	12 years	QALYs (utility values from literature and expert opinion)	£1144 per QALY
Fayter 2007^46^	Childhood screening	Short stature screening (no monitoring)	5 years	QALY-based CEA	Model based	Lifetime	QALYs (utility values from literature)	£9500 per QALY gained
Fortnum 2016^47^	Childhood screening	Hearing screening (no screening)	4–5 years	QALY-based CEA	Model based	4 years	QALYs (utility values from literature)	The SES programme is dominated
Grill 2006^48^	Childhood screening	Hospital hearing screening (community)	Newborn	Non-QALY-based CEA	Model based	10 years	Quality weighted detected child months	£2423 per detected child; £25 per quality weighed detected child month
Barber 2015^49^	Health promotion	Preschoolers in the Playground, PiP (usual care)	1–4 years	QALY-based CEA	Trial based	1 year	QALYs (EQ-5D and PedsQL)	£19 588 per QALY

*(Continued)*

**Table 1:** Continued.

Author year	Intervention category	Intervention (comparator)	Population	Type of evaluation	Study type	Time horizon	Outcomes captured (quality of life instrument)	Primary result of the evaluation
Hollingworth 2012^50^	Health promotion	Obesity/overweight interventions (no/minimal intervention)	4–5 years	Non-QALY-based CEA	Model based	Lifetime	Life years gained	£66 567 per life year gained (BMI SD score reduction of 0.03); £13 589 per life year gained (0.13 BMI SD score reduction)
Renwick 2018^51^	Health promotion	Smoking home intervention (usual care)	Under 5 years	Non-QALY-based CEA	Trial based	12 weeks	Average 16–24 h levels of particulate matter of < 2.5 μm diameter (PM_2.5_); the number of quitters	£131 per additional 10 μg/m^3^ reduction of 16–24 h PM2.5; £71 per additional quitter
Atkins 2012^52^	Health protection	RotaTeq (no vaccination)	Under 6 months	QALY-based CEA	Model based	50 years	QALYs (utility values from the literature)	Dynamic model: £27 133 per QALY. Static model: £34 728 per QALY. Other scenarios presented.
Baguelin 2015^53^	Health protection	LAIV (no vaccination)	2–4 years	QALY-based CEA	Model based	10 years	QALYs (utility values from the literature)	£2613 per QALY
Beck 2021^54^	Health protection	4CMenB vaccination (no vaccination)	Under 1 year	QALY-based CEA	Model based	100 years	QALYs (utility values from the literature)	£18 645 per QALY gained
Brisson 2003^55^	Health protection	VZV vaccination, infant strategy (no vaccination)	12–15 months	QALY-based CEA	Model based	80 years	QALYs (HUI-2)	VZV infant vaccination strategy is dominated
Christensen 2013^56^	Health protection	New ‘MenB’ vaccine (no vaccination)	2 months to 4 years	QALY-based CEA	Model based	100 years	QALYs (utility values from the literature)	Between £162 800 and £290 000 per QALY gained (cohort model); and between £91 800 and £97 600 per QALY (dynamic model)
Christensen 2014^57^	Health protection	Bexsero (no vaccination)	2 months to 1 year	QALY-based CEA	Model based	100 years	QALYs (EQ-5DY)	Ranged from £163 100 to £221 000 per QALY.
Edmunds 2002^58^	Health protection	Acellular pertussis booster (no vaccination)	4 years	Non-QALY-based CEA	Model based	lifetime	Life-years gained; general practitioner consultation; and hospitalization averted	Booster doses range from £8463 to £49 511 per life year gained from the health perspective; £2489 to £36 941 per life year gained from a societal perspective.
Hodgson 2020^59^	Health protection	RSV vaccination, MAB (status quo)	Under 5 years	QALY-based CEA	Model based	10 years	QALYs (EQ-5D)	Results presented as the maximum purchasing price per course for programmes to be cost-effective. For: MAB-VHR-S (£4342.97); MAB-HR-S (£201.15); MAD; MAB-HR-S+ (£87.03); VAC-INF-S (£94.76).
Jit 2007^60^	Health protection	Rotavirus vaccination (no vaccination)	2–4 months	QALY-based CEA and non-QALY-based CEA	Model based	Unclear	QALYs (HUI-2 and EQ-5D)	£79 905 per QALY gained; £525 per episode prevented; £3803 per hospitalization prevented (using RotaTeq). £60 928 per QALY gained; £391 per episode prevented; £3647 per hospitalization prevented (using Rotarix).
Jit 2009^61^	Health protection	Rotavirus vaccination	2–4 months	QALY-based CEA	Model based	5 years	QALYs (HUI-2 and EQ-5D)	The ICER is EUR110 000 per QALY gained (Rotarix vaccination programme) and EUR160 000 per QALY gained (RotaTeq vaccination programme)

*(Continued)*

**Table 1:** Continued.

Author year	Intervention category	Intervention (comparator)	Population	Type of evaluation	Study type	Time horizon	Outcomes captured (quality of life instrument)	Primary result of the evaluation
Jit 2010^62^	Health protection	Rotavirus vaccination (current care)	2–4 months	QALY-based CEA	Model based	5 years	QALYs (HUI-2 and EQ-5D)	EUR110 000 per QALY gained (Rotarix vaccination) and EUR150 000 per QALY gained (RotaTeq vaccination)
Knerer 2012^63^	Health protection	Pneumococcal vaccination (PCV-13)	Under 2 years	QALY-based CEA	Model based	94 years	QALYs (utility values from literature)	Pneumococcal conjugate vaccine dominates (positive QALYs, negative costs).
Lorgelly 2007^64^	Health protection	Rotavirus vaccination programme (no vaccination)	Newborn	Non-QALY-based CEA	Model based	5 years	Gastroenteritis episode avoided; GP visit avoid; hospitalization visit avoided; life years saved	£60.41 per episode avoided; £177 212 per life year saved.
Martin 2009^65^	Health protection	Rotarix (no vaccination)	Under 6 months	QALY-based CEA	Model based	Lifetime	QALYs (EQ-5D)	£23 298 per QALY
McIntosh 2003^66^	Health protection	Pneumococcal vaccination (no vaccination)	Under 6 months	Non-QALY-based CEA	Model based	10 years	Life years saved	£31 512 per life year saved
Melegaro 2004^67^	Health protection	Pneumococcal vaccination (no vaccination)	2 months to 2 years	Non-QALY-based CEA and QALY-based CEA	Model based	Lifetime	Life years gained; QALYs (utility values from literature)	£70 699 per life year gained; £31 021 per QALY
Pitman 2013^68^	Health protection	Influenza vaccination (current policy)	2–4 years	QALY-based CEA	Model based	200 years	QALYs (utility decrement from literature)	TIV in 2–4-year olds is dominated. LAIV in 2–4-year olds is cost saving
Siddiqui 2011^69^	Health protection	HBV programme (current vaccination practice)	Under 6 months	QALY-based CEA	Model based	99 years	QALYs (utility values from literature)	£263 000 per QALY (for universal infant vaccination programme); £90 000 per QALY (for the selective infant programme)
Thomas 2018^70^	Health protection	RSV vaccination (no vaccination)	Under 2 years	CBA	Cohort study	Lifetime	Costs	B/C ratios: 7.726 for bronchopulmonary dysplasia; 0.694 for congenital heart disease; 1.391 for extreme immaturity; 1.426 for premature babies; 0.465 for all other RSV admissions. All results for year of 2012/2013.
Trotter 2002^71^	Health protection	Meningitis C vaccination (no vaccination)	Under 4 years	Non-QALY-based CEA	Model based	Lifetime	Life years saved	0–4-month programme: £14 630 per life year saved; 5–11-month programme: £9493 per life year saved; 1–4 year programme: £5826 per life year saved
Trotter 2006a^72^	Health protection	Meningococcal vaccination (no vaccination)	Under 1 year	Non-QALY-based CEA and QALY-based CEA	Model based	100 years	Life years gained and QALYs (utility values from literature)	2–4-month programme: £38 164 per life year saved and £31 152 per QALY.

*(Continued)*

**Table 1:** Continued.

Author year	Intervention category	Intervention (comparator)	Population	Type of evaluation	Study type	Time horizon	Outcomes captured (quality of life instrument)	Primary result of the evaluation
Trotter 2006b^73^	Health protection	Meningococcal vaccination (current schedule)	Under 2 years	Non-QALY-based CEA	Model based	75 years	Life years gained	Strategy 2: £4 498 000 per life year; Strategy 3a: (2, 4, 13 months) –£ 2000 per life year gained; Strategy 3ab: (3, 13 months) –£4 811 000 per life year gained; Strategy 4: –£16 419 000 per life year gained
Achana 2016^74^	Injury prevention	Six intervention combinations of education, equipment, home inspection and fitting (usual care)	Under 4 years	QALY-based CEA and non-QALY-based CEA	Model based	100 years	QALYs (utility values from the literature) and numbers of poison cases avoided	Non-QALY-based CEA: lowest ICER was education at £2888 per poison avoided. QALY-based CEA: lowest ICER was education at £41 330 per QALY gained.
Kendrick 2017a^75^	Injury prevention	(i) Functional smoke alarm (usual care) (ii) Safe hot tap water temperature (usual care) (iii) Promoting safety gate possession and use (usual care) (iv) Promoting the safe storage of medicines (usual care) (v) Promoting the safe storage of household and other products (usual care)	Under 5 years	QALY-based CEA	Model based	100 years	QALYs (utility values from literature)	(i) Education + equipment is £34 200 per QALY gained. (ii) Education is £40 271 per QALY gained. (iii) Education is £284 068 per QALY gained. (iv) Education is £41 330 per QALY gained. (v) All interventions were more costly and less effective than usual care.
Kendrick 2017b^75^	Injury prevention	IPB with or without facilitation (usual care)	Under 3 years	Non-QALY-based CEA	Trial based	1 year	Probability of having a fire escape plan	Injury prevention briefing only: £1260 per additional fire escape plan, injury prevention briefing +£616.13 per additional fire escape plan
Phillips 2011^76^	Injury prevention	Scald prevention (waiting list)	Under 5 years	Non-QALY-based CEA and ROI (not stated)	Trial based	1 year	Risk reduction (scalds)	Scald prevention intervention: net savings of £7273 per scald avoided (NHS perspective), £53 949 per scald avoided (societal perspective). The benefit per £1 spent is £1.41 for an NHS perspective and (£0.47) for a lifetime perspective.
Saramago 2014^77^	Injury prevention	Fire injury prevention interventions (usual care)	Under 5 years	QALY-based CEA	Model based	100 years	QALYs (utility values from literature)	Non-dominated interventions: education plus low cost/free safety equipment, £34 200 per QALY gained; education plus low cost/free safety equipment plus fitting plus home inspection at £3 466 635 per QALY gained.
Bessey 2019^78^	Newborn screening	SCID screening (no screening)	Newborn	QALY-based CEA	Model based	5 years	QALYs (EQ-5D-3L)	£18 222 per QALY gained

*(Continued)*

**Table 1:** Continued.

Author year	Intervention category	Intervention (comparator)	Population	Type of evaluation	Study type	Time horizon	Outcomes captured (quality of life instrument)	Primary result of the evaluation
Bessey 2018^79^	Newborn screening	X-ALD screening (no screening)	Newborn	QALY-based CEA	Model based	Lifetime	QALYs (EQ-5D-5L)	Screening dominates (positive QALYs, negative costs)
Burke 2012^80^	Newborn screening	(i) Universal newborn hearing screening and (ii) one-stage universal screening (selective screening)	Newborn	Non-QALY-based CEA	Model based	Unclear	Cases detected	£36 181 per case detected.
Davies 2000^81^	Newborn screening	Neonatal screening nurse follow-up (targeted screening)	Newborn	Non-QALY-based CEA	Model based	Unclear	SCD cases identified	Range of ICERs reported for various disease incidence rates. For example, prevalence of 0.1 or 0.3 per 1000 births, results in ICERs in the range £25 000– £100 000 per case identified
Ewer 2012^82^	Newborn screening	Pulse oximetry screening (clinical examination)	Newborn	Non-QALY-based CEA	Model based	1 year	Detection of CHD	£24 900 per timely diagnosis
Griebsch 2007^83^	Newborn screening	Congenital heart defect screening (clinical examination)	Newborn	Non-QALY-based CEA	Model based	1 year	Timely diagnosis of life-threatening congenital heart defects	Pulse oximetry is £4894 per additional timely diagnosis; screening echocardiography £4 496 666 per additional timely diagnosis.
Knowles 2005^84^	Newborn screening	Congenital heart defect screening (clinical examination)	Newborn	Non-QALY-based CEA	Model based	1 year	Timely diagnosis	£4894 per timely diagnosis
Pandor 2004^36^ Pandor 2006^37^	Newborn screening	Inborn errors of metabolism screening (screening for PKU only)	Newborn	Non-QALY-based CEA	Model based	80 years	Life years gained; cases of inborn error of metabolism detected	–£7359 per case of inborn error of metabolism detected; ICER for cost per life year gained are not reported.
Roberts 2012^85^	Newborn screening	Congenital heart defect screening (clinical examination)	Newborn	Non-QALY-based CEA	Model based	1 year	Case of timely diagnosis	£24 900 per timely diagnosis of significant congenital heart defects
Simpson 2005^86^	Newborn screening	Cystic Fibrosis screening (no screening)	Newborn	QALY-based CEA	Model-based	Lifetime	QALYs (QWB)	£6864 per QALY
Uus 2006^87^	Newborn screening	Newborn Hearing Screening Programme (NHSP) (infant distraction test)	Newborn	Non-QALY-based CEA	Trial based	10 years	Cases detected	£12 527 per case detected
Davenport 2003^88^	Oral health	3-, 6-, 12-, 18-, 24- and 36-month dental check recall policies (unclear)	3 months	Non-QALY-based CEA	Model based	6 years	Number of teeth free from decay, fillings or extraction	No ICERs reported.
Davies 2003^89^	Oral health	The provision of free toothpaste and toothbrushes to 3 months (doing nothing)	1 year	Non-QALY-based CEA	Trial based	4 years	Decayed, missing and filled teeth reduction by one unit; child kept free of caries experience; child kept free of extraction experience	£80.83 per tooth saved from carious attack; £424.38 per child kept free of caries experience; £679.01 per extraction avoided

*(Continued)*

**Table 1:** Continued.

Author year	Intervention category	Intervention (comparator)	Population	Type of evaluation	Study type	Time horizon	Outcomes captured (quality of life instrument)	Primary result of the evaluation
Kay 2018^90^	Oral health	Supervised tooth brushing (no intervention)	5 years	QALY-based CEA	Model based	3 years	QALYs (utility values from literature)	Spending <£55 per child on supervised tooth brushing is cost-effective; spending <£100 on varnish would be cost-effective over 3 years
Kowash 2006^91^	Oral health	Out-reach education programme (unclear)	Under 1 year	CBA and non-QALY-based CEA	Trial based	3 years	Monetary and decayed, missing or filled tooth or tooth surface	The B/C ratio is 5.6. Cost-effectiveness ratio is 1.8.
O’Neill 2017^92^	Oral health	Caries prevention (advice only)	2–3 years	Non-QALY-based CEA	Trial based	3 years	Proportion caries free; number of carious surfaces; number of episodes of pain	£2092.59 per caries free person; £250.58 per carious surface; £259.07 per number of pain episodes
Tickle 2016^93^	Oral health	NIC-PIP caries prevention (prevention advice alone)	2–3 years	Non-QALY-based CEA	Trial based	3 years	Caries-free person; carious surfaces; episodes of pain	£2092.59 per proportion caries free; £250.58 per number of carious surfaces; £259.07 per episode of pain
Barnardo’s2012a^94^	Parenting support	Barnardo’s Children’s Centre Service: Stay and Play (unclear)	Under 2 years	SROI	Cohort study	5 years	Monetary outcomes	Approximately £2 for every £1 invested
Barnardo’s2012b^94^	Parenting support	Barnardo’s Children’s Centre Service: Family Support Worker (unclear)	Under 5 years	SROI	Cohort study	5 years	Monetary outcomes	£4.50 for every £1 invested
Edwards 2007^95^	Parenting support	The Webster-Stratton Incredible Years basic parenting programme (waiting list)	3–4 years	Non-QALY-based CEA	Trial based	1 year	ECBI-I	£71 per one point change in the ECBI-I score
Gardner 2017^96^	Parenting support	IY Basic parenting programme (no intervention)	5 years	Non-QALY-based CEA and ROI	Model based	25 years	ECBI-I	A WTP of £109 per point improvement on the ECBI-I is 50% probability of being cost-effective. In the ‘high-cost’ scenario, the ROI is ‘nearly fourfold’. Assumed to be an ROI of 4 for the results.
McAuley 2004^97^	Parenting support	Home Start support (no home start support)	Under 5 years	Non-QALY-based CEA and CCA	Cohort study	1 year	PSI; EPDS; RSE; BITSEAS; MSSI	The intervention was assumed to be dominated (no effect difference and increases costs in the Home Start arm)
Morell 2000a^35^ Morell 2000b^98^	Parenting support	Postnatal support from a community midwifery support worker (no support worker)	Newborn	CCA (not specified but reports costs and outcomes separately)	Trial based	6 months	SF-36; Duke functional social support; Edinburgh postnatal depression scale; number breastfeeding only; number formula milk feeding only	No evidence of differences in SF-36, Edinburgh postnatal depression scale, and Duke functional social support scale) and rates of breast feeding between the two groups. The difference in total NHS costs between the groups was £178.61.

*(Continued)*

**Table 1:** Continued.

Author year	Intervention category	Intervention (comparator)	Population	Type of evaluation	Study type	Time horizon	Outcomes captured (quality of life instrument)	Primary result of the evaluation
Simkiss 2013^99^	Parenting support	The Family Links Nurturing Programme (no screening)	2–4 years	QALY-based CEA	Trial based	10 years	QALYs (SF-6D, PedsQL)	£34 913 per QALY over 5 years and £18 954 per QALY over 10 years
Tudor Edwards 2016^100^	Parenting support	IY BASIC parenting programme (waiting list)	3–4 years	Non-QALY-based CEA	Trial based	6 months	SDQ; ECBI; APS	£1295 per one point improvement in SDQ; £237 per one point improvement in ECBI-I; £9477 per one point improvement in APS
Chance 2013^101^	Parenting support and health promotion	Cambridgeshire’s Funded Two-year-old Childcare (unclear)	2 years	SROI	Cohort study	5 years	Monetary outcomes	£8.40 for every £1 invested.
Mujica Mota 2006^102^	Parenting support and health promotion	Means-tested access to full-time or part-time day care at the Hackney Early Years Centre (childcare secured themselves)	6 months to 3.5 years	Non-QALY-based CEA	Trial based	18 months	Proportion of mothers in paid employment or education at 18 months	£38 550 per additional woman in paid employment; societal perspective shows it to be cost saving.
Barlow 2019^103^	Reducing risk of abuse/maltreatment	Parents under Pressure, PuP (treatment as usual)	Under 2 years	QALY-based CEA	Trial based	1 year	QALYs (EQ-5D-5L)	£34 095 per QALY (NHS and PSS perspective); £56 269 per QALY (societal perspective)
Boyd 2016^104^	Reducing risk of abuse/maltreatment	New Orleans-Glasgow model (existing Glasgow model)	Under 5 years	CCA	Cohort study	5 years	Probability of one and two episodes in care	Reduced probability of two episodes in care (incremental reduction of 0.41) and reduced mean cost per child in the model (incremental difference of £6820).

AOM, acute otitis media; APS, Arnold–O’Leary Parenting Scale; BC, breast cancer;; CHD, congenital heart defect; ECBI, Eyberg Child Behaviour Inventory; GI, Gastrointestinal infection; HUI, health utilities index; ICER, incremental cost-effectiveness ratio; LRTI, lower respiratory tract infection; NEC, necrotizing enterocolitis; PedsQL, paediatric quality of life inventory; QWB, quality of well-being; SDQ; strengths and difficulties questionnaire; SF-6D, short-form 36 health survey.

A little under half of the evaluations were QALY-based CEAs (46%; 32/69). The majority of evaluations were non-QALY-based CEAs (49%; 34/69), with the health outcomes used including life years gained/saved (29%; 10/34), oral health outcomes such as dental caries detected or number of teeth free from decay (15%, 5/34) and cases of a specific disease or condition detected (12%, 4/34). See Table 2 for the list of outcomes. One evaluation (1%) was a CCA^104^ and one (1%) a CCA alongside a CEA.^97^ Of the studies reporting outcomes in monetary terms, the evaluations identified were SROI (4%; 3/69), CBA (3%; 2/69) with one of the CBAs being conducted alongside a CEA. Finally, four (4%) were not explicit about the type of evaluation used; however, detailed inspecting suggested two of them could be classified as CCA^35^^,^^42^^,^^98^ and two as ROI analysis.^76^^,^^96^

**Table 2:** Summary of approaches

	Category	Total	%
Type of evaluation^†^	QALY-based CEA	32/77	42
	Non-QALY-based CEA	34/77	44
	CCA	4/77	5
	CBA	2/77	3
	SROI	3/77	4
	ROI	2/77	1
Outcomes used in CEA	Life years	10/34	29
	Multiple oral health outcomes	5/34	15
	Cases detected	4/34	12
	ECBI-I	3/34	9
	Timely diagnosis	3/34	9
	Multiple breastfeeding outcomes	2/34	6
	Poison cases avoided	1/34	3
	Quality weighted detected child months	1/34	3
	Probability of having a fire escape plan	1/34	3
	Proportion of mothers in paid employment or education at 18 months	1/34	3
	Risk reduction (scalds)	1/34	3
	PM2.5 level and the number of quitters	1/34	3
	Unclear	1/34	3
Perspective	NHS or NHS and PSS	38/69	55
	Societal	9/69	13
	NHS (scenario analysis of societal)	9/69	13
	Public sector	6/69	9
	NHS, education services, patients and family	1/69	1
	Public payer	1/69	1
	Health and other public sector providers	1/69	1
	NHS and the family	1/69	1
	NHS and ‘other government departments’	1/69	1
	Children and their families	1/69	1
	NHS (includes scenario with lost labour costs to families of children)	1/69	1
Incorporation of equity considerations in analysis?	Yes	2/69	3
	No	67/69	97
Use of decision modelling?	Yes	46/69	67
	No	23/69	33

^†^For the purpose of the summary of results, the number of evaluations was considered to be 77 as eight evaluations presented the results of two types of evaluation.

ECBI, Eyberg Child Behaviour Inventory; PM, particulate matter.

The most commonly reported perspective was the NHS or NHS and personal social services (PSS) (55%, 38/69), which is consistent with the latest NICE methods guidance.^11^ A considerable number were defined as having a societal perspective in the base case (13%; 9/69) or presenting a societal perspective alongside an NHS or NHS and PSS perspective (13%; 9/69). The breadth of the incorporated costs and outcomes in the societal perspectives, however, differed across evaluations. These included costs borne by the family/caregiver (72%; 13/18), lost wages (61%, 11/18), household expenditure (28%, 5/18), travel time (11%, 2/18), the local authority/council (22%, 4/18), legal costs (11%, 2/18) and education costs (17%; 3/18). The outcomes captured in the societal perspective were solely health in 11 evaluations (61%; 11/18) including QALYs (33%; 6/18) or another measure of health (28%; 5/18). Outcomes were captured in monetary units in four evaluations with a societal perspective (22%; 4/18). See Supplementary Material, Supplementary Appendix 5 for further details.

The time horizons over which the costs and outcomes of the interventions were captured were predominantly one of two categories: those with a short time horizon, i.e. 0–10 years (54%, 37/69), or those with a lifetime horizon that was categorized as 76 years and over (32%, 22/69). The exact number of years over which the costs and outcomes were evaluated was unclear in the case of four evaluations (6%; 4/69) and in the evaluation by Pokhrel,^42^ the time horizon differed according to the type of outcome evaluated.

Decision analytic modelling was used in the majority of evaluations (67%; 46/69). Of these, the adopted approaches were decision trees (28%; 13/46), dynamic transmission models (22%; 10/46), Markov models (20%; 6/46) and a decision tree followed by a Markov model (4%; 2/46). Five economic evaluations (11%) described the modelling approach as a ‘cohort model’, but the exact approach was unclear. Four evaluations (9%) were based on decision models, yet little information on the approach was provided. See Supplementary material, Supplementary Appendix 5 for further information.

The overwhelming majority of evaluations did not formally incorporate equity considerations (97%; 67/69), see Table 2. Two evaluations (3%)^74^^,^^88^ considered the cost-effectiveness results across two different social groups. This provided insight into the distribution of health-related outcomes and therefore the cost-effectiveness across social groups.

### Reported value for money of the interventions

**Fig. 2:**
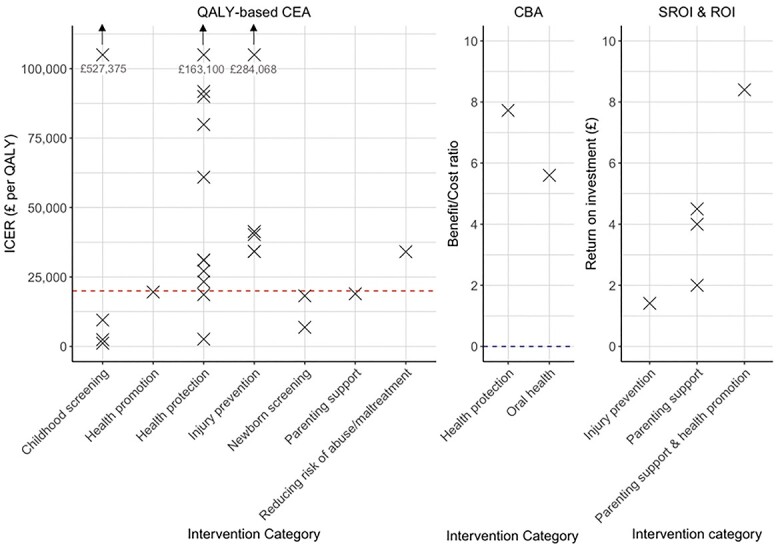
Results of the QALY-based CEAs, CBAs and SROI and ROI by intervention category.

Figure 2 presents the reported results from each study, grouped by the framework used and the intervention group. For QALY-based CEAs, 26 evaluations reported an incremental cost per QALY result. In Fig. 2, the NICE-adopted policy threshold of £20 000 per QALY is represented by the dashed red line in the QALY-based CEA plot. Six additional interventions were not included in Fig. 2: three were considered to be dominant and three were considered to be dominated. See Table 1 for further details. Two evaluations presented the results in Euros per QALYs and were therefore excluded from Fig. 2. The results of the non-QALY-based CEAs and the CCAs are not included as the outcomes of the results differ across evaluations.

Both of the CBAs reported a benefit to cost ratios above 0 (the point at which the intervention is considered value for money). The study by Thomas^70^ presented the results by disease category, only those results for bronchopulmonary dysplasia are included in Fig. 2 as this disease had the highest benefit/cost (B/C) ratio. The other disease category B/C ratios reported in Thomas can be seen in Table 1. All of the identified SROIs and ROIs indicated that for every pound spent on the intervention, a return of >£1 would be generated (Fig. 2). For four of the five SROIs and ROIs, the evaluations were conducted without a comparator arm.

### Quality assessment

Fourteen papers reported on all aspects of the CHEERS Checklist. In the majority of the categories of the Checklist, reporting was good, including the perspective, time horizon and competing alternatives. However, the value of the reported costs and outcomes was unclear or not reported in 16 evaluations, with 20 of the evaluations with a time horizon over 1 year failing to report the discount rate for costs and outcomes.

Finally, only 29 of the evaluations covered all of the issues of concern in the presentation and discussion of results. The results of the detailed CHEERS checklist are presented in Supplementary Material, Supplementary Appendix 3.

## Discussion

The results show the breadth of UK-focussed economic evaluations of early childhood public health interventions reported or discussed in the published literature. The methods adopted in the demonstration of value for money showed a lack of consistency across many aspects including the type of economic evaluation, the health outcomes captured and the perspective adopted. Fourteen papers reported on all aspects of the CHEERS Checklist meaning 55 (80%) were lacking elements required of a well-reported economic evaluation.

Many of the evaluated interventions were deemed value for money from the perspectives taken. Twelve (38%) of the QALY-based CEAs are cost-effective against the NICE policy threshold of £20 000 per QALY. However, the interpretation of some of the results may require particular consideration given the health, economic, political and social context of these studies may have changed between 2000 and present day.

Because of the evaluative framework chosen for many of the other studies a robust statement of value for money of the intervention is not always possible. For example, in non-QALY-based CEAs, an explicit statement of cost-effectiveness is challenging when the outcome is a metric other than a generic measure of health such as the QALY as it is not possible to compare across different health dimensions. Much has been made of the limitations and challenges of using QALYs for paediatric populations^17^^,^^19^ but their use does allow the comparison of interventions across diseases areas as well as the consideration of the displaced resources (or the ‘opportunity cost’).

All of the interventions evaluated using SROI, ROI or CBA frameworks could be considered value for money as they were deemed to generate more monetary benefits than the costs (having a ratio of >£1 of benefit per £1 of cost in the case of the SROI and ROI). Yet, caution is required when considering these results. None of the SROI or ROI evaluations incorporated the opportunity cost and it was made explicit in only one CBA.^70^ The exclusion of such a fundamental aspect of economic evaluations results in an overestimation of the value of the intervention and risks doing more harm than good to the public by neglecting the health foregone through the net effect of spending. Furthermore, four out of five of the SROI and ROI evaluations were conducted without a comparator. The lack of the inclusion of the opportunity cost or a comparator may feed into the previously reported challenges of allocation decision using ROI.^105^

The broad range of the types of evaluation and outcomes may reflect the diverse nature and needs of the decision-makers relevant to such interventions. Public health commissioning decisions in the UK are often the responsibility of local commissioners of services, such as local authorities and clinical commissioning groups (CCGs), not national decision-makers such as NICE. Although NICE’s public health approach allows for flexibility in the methods, evaluations conducted using the NICE methods guide may fall short of reflecting the challenges faced by CCGs.^106^

Although only a minority, a number of evaluations attempted to incorporate the wider social value of the intervention beyond the value to the health care system. A total of 18 evaluations adopted a ‘societal perspective’ but the results identified a lack of consistency in the included aspects of value. The inclusion of lost productivity to the parent or caregiver (in the form of wages lost) featured heavily in the evaluations, as did incorporating costs falling on special education services and legal services, yet none featured consistently. The implication of such inconsistencies is that value judgements about what ‘should’ count are falling on the researchers rather than socially legitimate decision-makers.^107^ Public health guidance issued by NICE^108^ does allow for flexibility in the costs and outcomes considered in an economic evaluation, but the lack of explicit value judgements may facilitate such inconsistencies.

The results showed the most common time horizons were either 0–5 years or those that extended beyond 76 years. Reasons for this appear to be based around whether an intervention was a trial-based evaluation or those that incorporated decision modelling to model the long-term costs and outcomes. Guidance in the economic evaluation literature indicates that time horizons should be long enough to reflect all of the important differences in costs and outcomes between comparators.^10^^,^^11^ Such horizons may be well defined for patient-focussed health technologies but not for population-focussed interventions that aim to change behaviour, education, housing and so on. Given the evidence linking the social determinants of health and life expectancy,^109^ it stands that a lifetime horizon may be more appropriate.

One aspect of relative consistency in the methods was the lack of the formal incorporation of equity considerations. Interventions implemented in early life have considerable potential to disrupt existing inequalities^7^ and remain a fundamental reason for targeting these important years. Yet, the formal incorporation of equity does not appear to be common practice in economic evaluation in this setting. There are now a number of approaches to formally incorporate equity considerations into CEAs.^110^

The focus of this review was to identify interventions relevant to UK decision-makers. However, there may be important information available in an international context to aid learnings around the use of methods and approaches relevant to the UK. Future research may consider describing the methods and approaches adopted in the global evidence base to highlight consistencies in the demonstration of value for money in those economic evaluations developed for an international context.

### Limitations

A limitation is that there may be relevant and uncaptured evaluations in the grey literature. This is evidenced through the identification of evaluations produced by NICE,^38^ Social Value UK,^101^ Barnardo’s^94^ and the Joseph Rowntree Foundation,^97^ which were not identified in the database search but rather through the literature reviews. The search strategy included the HMIC database and lists grey literature amongst its coverage, hence the decision to include relevant grey literature in the reference searches of systematic reviews. The inclusion of the grey literature identified though the supplementary reference searching does in part explain the high number of studies identified in this way (5 of the 13). We considered it important to include grey literature as it is included in the HEER tool,^33^ yet a pragmatic decision was made for the purpose of this review. Future literature reviews may consider searching and identifying a wider range of grey literature sources.

A further limitation was the difficulty posed in defining ‘public health’ for the purpose of the search strategy. The review focussed on interventions that aimed to improve the health of the infant or child yet health improvement in early childhood may be dependent on lifestyle and environment not merely based on biology and genetics.^6^ It stands that a social model of health may have generated different results. A pragmatic decision was made to include health terms and terms to capture the wider determinants of health in the search strategy.

## Conclusion

In addition to identifying the breadth of evidence available in the published literature, this review provides an overview of the inconsistent methodological approaches used. The lack of consistency identified in the methods has highlighted a number of issues that may require consideration in the future generation of economic evaluations of similar interventions to aid decision-making. It is hoped the results can provide a foundation to help improve decision-making and provide a starting point for methodological developments in the early childhood public health context.

## Supplementary Material

PM_BMB_Supplementary_Material_ldac035

## Data Availability

The data underlying this article are available in the article and in its online supplementary material.
