# Supplementary material for: Methods of assessing value for money of UK-based early childhood public health interventions: a systematic literature review
Source: Br Med Bull. 2022 Dec 19;145(1):88–109. doi: 10.1093/bmb/ldac035 (PMC10075243; doi:10.1093/bmb/ldac035)
Supplement: PM_BMB_Supplementary_Material_ldac035 [file pm_bmb_supplementary_material_ldac035.docx]

**Supplementary Material**

**Appendix 1: Search Strategies**

**Ovid MEDLINE(R) ALL <1946 to August 13, 2021>**

via Ovid http://ovidsp.ovid.com/

Date range searched: 1946 to 13^th^ August 2021

Date searched: 16^th^ August 2021

Records retrieved: 2408

The MEDLINE strategy below includes the CADTH economics search filter for Ovid Medline (lines 52-74) and the NICE UK search filter for Ovid Medline (lines 80-90).

Economic Evaluations/Cost/Economic Models - Ovid Medline. Strings attached: CADTH database search filters [Internet]. Ottawa: CADTH; 2016. [Accessed: 16 August 2021]. Available from: https://www.cadth.ca/resources/finding-evidence/strings-attached-cadths-database-search-filters#health

Ayiku L, Levay P, Hudson T, Craven J, Barrett E, Finnegan A and Adams R. The MEDLINE UK filter: development and validation of a geographic search filter to retrieve research about the UK from OVID MEDLINE. Health Information and Libraries Journal, 2017 34 (3): 200-216. (Publisher: Wiley. © 2017 Crown copyright. Health Information and Libraries Journal © 2017 Health Libraries Group.)

1 exp Pediatrics/ (60711)

2 exp Child/ (1996653)

3 exp Infant/ (1181492)

4 exp Infant, Newborn/ (631896)

5 exp Infant, Low Birth Weight/ (36085)

6 exp Infant, Very Low Birth Weight/ (10753)

7 exp Infant, Premature/ (58941)

8 (p?ediatric* or child* or preemie* or baby or babies or infant* or toddler* or neo nat* or neo-nat* or neonat* or newborn* or new-born* or newly born* or newly-born* or preschool* or pre-school* or schoolchild* or school-child* or schoolboy* or school-boy* or schoolgirl* or school-girl* or school-age* or prekindergarten or pre-kindergarten or kindergarten or boy* or girl* or kid* or LBW or VLBW or ELBW or "low birth weight").ti,ab. (2791606)

9 (under adj (five* or "5") adj2 (age* or old*)).ti,ab. (2226)

10 ("birth to 5" or "birth to five").ti,ab. (1388)

11 or/1-10 (3825111)

12 Public Health/ (86906)

13 Health Promotion/ (77265)

14 Health Literacy/ (7074)

15 Health Education/ (62112)

16 "Social Determinants of Health"/ (4516)

17 Child Health/ (3726)

18 Child Development/ (48301)

19 Child Guidance/ (892)

20 Child Welfare/ (22213)

21 Child Abuse/ (23367)

22 Child Nutrition Disorders/ (3672)

23 Infant Health/ (1020)

24 Infant Welfare/ (2777)

25 exp Family Relations/ (96657)

26 Early Intervention, Educational/ (3231)

27 Early Medical Intervention/ (3313)

28 exp Physical Fitness/ (32981)

29 Diet, Healthy/ (5352)

30 Oral Health/ (18053)

31 ((early or early-years or early-life*) adj3 (program* or interven* or scheme* or initiative*)).ti,ab. (46348)

32 ((health* or wellness or welfare or well-being or wellbeing or safety or immuni*) adj5 (program* or interven* or scheme* or initiative* or encourag* or promot* or educat* or literacy or campaign* or improve* or improving)).ti,ab. (463672)

33 ((lifestyle* or diet* or food* or nutrition*) adj3 (intervention* or program* or chang* or modif* or improv* or enhanc* or adapt* or target* or alter* or impact*)).ti,ab. (159406)

34 ((behavio?r* or positive* or success*) adj3 (intervention* or program* or chang* or modif* or improv* or enhanc* or adapt* or impact*)).ti,ab. (251915)

35 ("best start in life" or "healthy child programme" or "healthy start programme" or "change for children").ti,ab. (543)

36 ((increas* or improv* or encourag* or support* or promot* or influen* or recommend* or motivat* or incentiv* or market* or advert* or subsid* or reward* or persua* or convinc* or instigat* or invest or benefit* or uptak* or start*) adj4 ((active or physically-active or health*) adj3 (living or life*))).ti,ab. (10533)

37 ((increas* or improv* or encourag* or support* or promot* or influen* or recommend* or motivat* or incentiv* or market* or advert* or subsid* or reward* or persua* or convinc* or instigat* or invest or benefit* or uptak* or start*) adj4 (exercise* or exercising or fitness)).ti,ab. (80371)

38 ((decreas* or minimis* or reduc* or discourag* or disincentiv* or dissuade* or deter* or prevent* or avert* or divert) adj4 (physical* adj2 inactiv*)).ti,ab. (449)

39 ((decreas* or minimis* or reduc* or discourag* or disincentiv* or dissuade* or deter* or prevent* or avert* or divert) adj4 ((inactiv* or unhealthy) adj3 (living or life*))).ti,ab. (138)

40 ((increas* or improv* or encourag* or support* or promot* or influen* or recommend* or motivat* or incentiv* or market* or advert* or subsid* or reward* or persua* or convinc* or instigat* or invest or benefit* or uptak* or start*) adj4 ((weight or body mass or BMI) adj2 (healthy or manage* or control* or loss* or loos* or decreas* or reduc*))).ti,ab. (22088)

41 ((decreas* or minimis* or reduc* or discourag* or disincentiv* or dissuade* or deter* or prevent* or avert* or divert) adj4 (obese or obesity or overweight)).ti,ab. (33832)

42 ((increas* or improv* or encourag* or support* or promot* or influen* or recommend* or motivat* or incentiv* or market* or advert* or subsid* or reward* or persua* or convinc* or instigat* or invest or benefit* or uptak* or start*) adj4 ((health* or balanced) adj2 (diet* or eating or food or nutrition*))).ti,ab. (9208)

43 ((increas* or improv* or encourag* or support* or promot* or influen* or recommend* or motivat* or incentiv* or market* or advert* or subsid* or reward* or persua* or convinc* or instigat* or invest or benefit* or uptak* or start*) adj4 ((salt or sugar or calorie*) adj2 (less or lessen or reduc* or restrict*))).ti,ab. (1591)

44 ((decreas* or minimis* or reduc* or discourag* or disincentiv* or dissuade* or deter* or prevent* or avert* or divert) adj4 ((fizzy or sugary or sweetened) adj2 (drink or beverage*))).ti,ab. (422)

45 ((breastfeed* or feeding) adj3 (advice or advis* or educat* or support*)).ti,ab. (5385)

46 ((decreas* or minimis* or reduc* or discourag* or disincentiv* or dissuade* or deter* or prevent* or avert* or divert) adj4 (malnutrition or malnourish* or undernourish* or overnutrition)).ti,ab. (3902)

47 ((increas* or improv* or encourag* or support* or promot* or influen* or recommend* or motivat* or incentiv* or market* or advert* or subsid* or reward* or persua* or convinc* or instigat* or invest or benefit* or uptak* or start*) adj4 ((oral* or dental*) adj2 (health* or care or hygien*))).ti,ab. (10345)

48 ((decreas* or minimis* or reduc* or discourag* or disincentiv* or dissuade* or deter* or prevent* or avert* or divert) adj4 ((oral* or dental* or tooth or teeth) adj2 (decay* or disease*))).ti,ab. (1945)

49 ((identif* or detect* or prevent*) adj3 ((domestic* or spousal or child* or caregiver* or care-giver* or parent* or maternal* or paternal* or physical* or emotional*) adj2 (neglect* or abuse* or abusive or violen* or harm or maltreat* or mistreat*))).ti,ab. (2577)

50 or/12-49 (1317434)

51 11 and 50 (370070)

52 Economics/ (27358)

53 exp "Costs and Cost Analysis"/ (248164)

54 Economics, Nursing/ (4005)

55 Economics, Medical/ (9147)

56 Economics, Pharmaceutical/ (3009)

57 exp Economics, Hospital/ (25259)

58 Economics, Dental/ (1919)

59 exp "Fees and Charges"/ (30838)

60 exp Budgets/ (13870)

61 budget*.ti,ab,kf. (31899)

62 (economic* or cost or costs or costly or costing or price or prices or pricing or pharmacoeconomic* or pharmaco-economic* or expenditure or expenditures or expense or expenses or financial or finance or finances or financed).ti,kf. (247110)

63 (economic* or cost or costs or costly or costing or price or prices or pricing or pharmacoeconomic* or pharmaco-economic* or expenditure or expenditures or expense or expenses or financial or finance or finances or financed).ab. /freq=2 (321647)

64 (cost* adj2 (effective* or utilit* or benefit* or minimi* or analy* or outcome or outcomes)).ab,kf. (178877)

65 (value adj2 (money or monetary)).ti,ab,kf. (2636)

66 exp models, economic/ (15742)

67 economic model*.ab,kf. (3633)

68 markov chains/ (15176)

69 markov.ti,ab,kf. (24797)

70 monte carlo method/ (29988)

71 monte carlo.ti,ab,kf. (53152)

72 exp Decision Theory/ (12550)

73 (decision* adj2 (tree* or analy* or model*)).ti,ab,kf. (28075)

74 or/52-73 (788188)

75 (return adj3 investment*).tw. (2469)

76 (SROI or ROI).tw. (11279)

77 or/75-76 (13350)

78 74 or 77 (799559)

79 51 and 78 (23175)

80 exp United Kingdom/ (377664)

81 (national health service* or nhs*).ti,ab,in. (225352)

82 (english not ((published or publication* or translat* or written or language* or speak* or literature or citation*) adj5 english)).ti,ab. (41374)

83 (gb or "g.b." or britain* or (british* not "british columbia") or uk or "u.k." or united kingdom* or (england* not "new england") or northern ireland* or northern irish* or scotland* or scottish* or ((wales or "south wales") not "new south wales") or welsh*).ti,ab,jw,in. (2210806)

84 (bath or "bath's" or ((birmingham not alabama*) or ("birmingham's" not alabama*) or bradford or "bradford's" or brighton or "brighton's" or bristol or "bristol's" or carlisle* or "carlisle's" or (cambridge not (massachusetts* or boston* or harvard*)) or ("cambridge's" not (massachusetts* or boston* or harvard*)) or (canterbury not zealand*) or ("canterbury's" not zealand*) or chelmsford or "chelmsford's" or chester or "chester's" or chichester or "chichester's" or coventry or "coventry's" or derby or "derby's" or (durham not (carolina* or nc)) or ("durham's" not (carolina* or nc)) or ely or "ely's" or exeter or "exeter's" or gloucester or "gloucester's" or hereford or "hereford's" or hull or "hull's" or lancaster or "lancaster's" or leeds* or leicester or "leicester's" or (lincoln not nebraska*) or ("lincoln's" not nebraska*) or (liverpool not (new south wales* or nsw)) or ("liverpool's" not (new south wales* or nsw)) or ((london not (ontario* or ont or toronto*)) or ("london's" not (ontario* or ont or toronto*)) or manchester or "manchester's" or (newcastle not (new south wales* or nsw)) or ("newcastle's" not (new south wales* or nsw)) or norwich or "norwich's" or nottingham or "nottingham's" or oxford or "oxford's" or peterborough or "peterborough's" or plymouth or "plymouth's" or portsmouth or "portsmouth's" or preston or "preston's" or ripon or "ripon's" or salford or "salford's" or salisbury or "salisbury's" or sheffield or "sheffield's" or southampton or "southampton's" or st albans or stoke or "stoke's" or sunderland or "sunderland's" or truro or "truro's" or wakefield or "wakefield's" or wells or westminster or "westminster's" or winchester or "winchester's" or wolverhampton or "wolverhampton's" or (worcester not (massachusetts* or boston* or harvard*)) or ("worcester's" not (massachusetts* or boston* or harvard*)) or (york not ("new york*" or ny or ontario* or ont or toronto*)) or ("york's" not ("new york*" or ny or ontario* or ont or toronto*))))).ti,ab,in. (1534733)

85 (bangor or "bangor's" or cardiff or "cardiff's" or newport or "newport's" or st asaph or "st asaph's" or st davids or swansea or "swansea's").ti,ab,in. (61019)

86 (aberdeen or "aberdeen's" or dundee or "dundee's" or edinburgh or "edinburgh's" or glasgow or "glasgow's" or inverness or (perth not australia*) or ("perth's" not australia*) or stirling or "stirling's").ti,ab,in. (226780)

87 (armagh or "armagh's" or belfast or "belfast's" or lisburn or "lisburn's" or londonderry or "londonderry's" or derry or "derry's" or newry or "newry's").ti,ab,in. (28975)

88 or/80-87 (2777779)

89 (exp africa/ or exp americas/ or exp antarctic regions/ or exp arctic regions/ or exp asia/ or exp australia/ or exp oceania/) not (exp United Kingdom/ or europe/) (3062526)

90 88 not 89 (2639366)

91 79 and 90 (2754)

92 exp animals/ not humans/ (4873476)

93 91 not 92 (2741)

94 limit 93 to yr="2000-Current" (2427)

95 remove duplicates from 94 (2408)

**Key:**

/ = indexing term (Medical Subject Heading: MeSH)

exp = exploded indexing term (MeSH)

? = replaces 0 or 1 character

* = truncation

ti,ab,tw,kf = terms in either title, abstract, textword or keyword heading word fields

jw,in = terms in either journal word or institution fields

adj3 = terms within three words of each other (any order)

**Embase <1974 to 2021 August 17>**

via Ovid http://ovidsp.ovid.com/

Date range searched: 1974 to 17^th^ August 2021

Date searched: 18^th^ August 2021

Records retrieved: 4120

The Embase strategy below includes the CADTH economics search filter for Ovid Embase (lines 50-68) and the NICE UK search filter for Ovid Embase (lines 74-84).

Economic Evaluations/Cost/Economic Models - Ovid Embase. Strings attached: CADTH database search filters [Internet]. Ottawa: CADTH; 2016. [Accessed: 16 August 2021].

Available from: https://www.cadth.ca/resources/finding-evidence/strings-attached-cadths-database-search-filters#health

Ayiku L, Levay P, Hudson T, Craven J, Finnegan A, Adams R and Barrett E. The Embase UK filter: validation of a geographic search filter to retrieve research about the UK from OVID Embase. Health Information and Libraries Journal, 2019 36 (2): 121-133. (Publisher: Wiley. © 2019 Health Libraries Group)

1 exp pediatrics/ (113669)

2 exp child/ (2769786)

3 exp infant/ (1033796)

4 exp newborn/ (551140)

5 exp low birth weight/ (66137)

6 exp very low birth weight/ (15959)

7 prematurity/ (110110)

8 (p?ediatric* or child* or preemie* or baby or babies or infant* or toddler* or neo nat* or neo-nat* or neonat* or newborn* or new-born* or newly born* or newly-born* or preschool* or pre-school* or schoolchild* or school-child* or schoolboy* or school-boy* or schoolgirl* or school-girl* or school-age* or prekindergarten or pre-kindergarten or kindergarten or boy* or girl* or kid* or LBW or VLBW or ELBW or "low birth weight").ti,ab. (3506590)

9 (under adj (five* or "5") adj2 (age* or old*)).ti,ab. (2821)

10 ("birth to 5" or "birth to five").ti,ab. (1823)

11 or/1-10 (4338575)

12 public health/ (196115)

13 exp health promotion/ (105259)

14 health literacy/ (13958)

15 health education/ (99558)

16 "social determinants of health"/ (11058)

17 child health/ (30025)

18 child development/ (46734)

19 child welfare/ (15505)

20 child abuse/ (31499)

21 exp child nutrition/ (110631)

22 infant welfare/ (1684)

23 exp child parent relation/ (89587)

24 exp early childhood intervention/ (3002)

25 early intervention/ (27619)

26 fitness/ (38503)

27 healthy diet/ (4731)

28 tooth disease/ (32918)

29 ((early or early-years or early-life*) adj3 (program* or interven* or scheme* or initiative*)).ti,ab. (68533)

30 ((health* or wellness or welfare or well-being or wellbeing or safety or immuni*) adj5 (program* or interven* or scheme* or initiative* or encourag* or promot* or educat* or literacy or campaign* or improve* or improving)).ti,ab. (587288)

31 ((lifestyle* or diet* or food* or nutrition*) adj3 (intervention* or program* or chang* or modif* or improv* or enhanc* or adapt* or target* or alter* or impact*)).ti,ab. (213174)

32 ((behavio?r* or positive* or success*) adj3 (intervention* or program* or chang* or modif* or improv* or enhanc* or adapt* or impact*)).ti,ab. (329737)

33 ("best start in life" or "healthy child programme" or "healthy start programme" or "change for children").ti,ab. (692)

34 ((increas* or improv* or encourag* or support* or promot* or influen* or recommend* or motivat* or incentiv* or market* or advert* or subsid* or reward* or persua* or convinc* or instigat* or invest or benefit* or uptak* or start*) adj4 ((active or physically-active or health*) adj3 (living or life*))).ti,ab. (14185)

35 ((increas* or improv* or encourag* or support* or promot* or influen* or recommend* or motivat* or incentiv* or market* or advert* or subsid* or reward* or persua* or convinc* or instigat* or invest or benefit* or uptak* or start*) adj4 (exercise* or exercising or fitness)).ti,ab. (106659)

36 ((decreas* or minimis* or reduc* or discourag* or disincentiv* or dissuade* or deter* or prevent* or avert* or divert) adj4 (physical* adj2 inactiv*)).ti,ab. (590)

37 ((decreas* or minimis* or reduc* or discourag* or disincentiv* or dissuade* or deter* or prevent* or avert* or divert) adj4 ((inactiv* or unhealthy) adj3 (living or life*))).ti,ab. (173)

38 ((increas* or improv* or encourag* or support* or promot* or influen* or recommend* or motivat* or incentiv* or market* or advert* or subsid* or reward* or persua* or convinc* or instigat* or invest or benefit* or uptak* or start*) adj4 ((weight or body mass or BMI) adj2 (healthy or manage* or control* or loss* or loos* or decreas* or reduc*))).ti,ab. (33329)

39 ((decreas* or minimis* or reduc* or discourag* or disincentiv* or dissuade* or deter* or prevent* or avert* or divert) adj4 (obese or obesity or overweight)).ti,ab. (48320)

40 ((increas* or improv* or encourag* or support* or promot* or influen* or recommend* or motivat* or incentiv* or market* or advert* or subsid* or reward* or persua* or convinc* or instigat* or invest or benefit* or uptak* or start*) adj4 ((health* or balanced) adj2 (diet* or eating or food or nutrition*))).ti,ab. (11729)

41 ((increas* or improv* or encourag* or support* or promot* or influen* or recommend* or motivat* or incentiv* or market* or advert* or subsid* or reward* or persua* or convinc* or instigat* or invest or benefit* or uptak* or start*) adj4 ((salt or sugar or calorie*) adj2 (less or lessen or reduc* or restrict*))).ti,ab. (2069)

42 ((decreas* or minimis* or reduc* or discourag* or disincentiv* or dissuade* or deter* or prevent* or avert* or divert) adj4 ((fizzy or sugary or sweetened) adj2 (drink or beverage*))).ti,ab. (511)

43 ((breastfeed* or feeding) adj3 (advice or advis* or educat* or support*)).ti,ab. (6670)

44 ((decreas* or minimis* or reduc* or discourag* or disincentiv* or dissuade* or deter* or prevent* or avert* or divert) adj4 (malnutrition or malnourish* or undernourish* or overnutrition)).ti,ab. (5596)

45 ((increas* or improv* or encourag* or support* or promot* or influen* or recommend* or motivat* or incentiv* or market* or advert* or subsid* or reward* or persua* or convinc* or instigat* or invest or benefit* or uptak* or start*) adj4 ((oral* or dental*) adj2 (health* or care or hygien*))).ti,ab. (10903)

46 ((decreas* or minimis* or reduc* or discourag* or disincentiv* or dissuade* or deter* or prevent* or avert* or divert) adj4 ((oral* or dental* or tooth or teeth) adj2 (decay* or disease*))).ti,ab. (2094)

47 ((identif* or detect* or prevent*) adj3 ((domestic* or spousal or child* or caregiver* or care-giver* or parent* or maternal* or paternal* or physical* or emotional*) adj2 (neglect* or abuse* or abusive or violen* or harm or maltreat* or mistreat*))).ti,ab. (2992)

48 or/12-47 (1849133)

49 11 and 48 (526075)

50 Economics/ (241804)

51 Cost/ (59613)

52 exp Health Economics/ (895063)

53 Budget/ (30775)

54 budget*.ti,ab,kw. (42271)

55 (economic* or cost or costs or costly or costing or price or prices or pricing or pharmacoeconomic* or pharmaco-economic* or expenditure or expenditures or expense or expenses or financial or finance or finances or financed).ti,kw. (304827)

56 (economic* or cost or costs or costly or costing or price or prices or pricing or pharmacoeconomic* or pharmaco-economic* or expenditure or expenditures or expense or expenses or financial or finance or finances or financed).ab. /freq=2 (451083)

57 (cost* adj2 (effective* or utilit* or benefit* or minimi* or analy* or outcome or outcomes)).ab,kw. (251602)

58 (value adj2 (money or monetary)).ti,ab,kw. (3586)

59 Statistical Model/ (166664)

60 economic model*.ab,kw. (5409)

61 Probability/ (120621)

62 markov.ti,ab,kw. (32667)

63 monte carlo method/ (43799)

64 monte carlo.ti,ab,kw. (54829)

65 Decision Theory/ (1782)

66 Decision Tree/ (15443)

67 (decision* adj2 (tree* or analy* or model*)).ti,ab,kw. (39658)

68 or/50-67 (1747244)

69 (return adj3 investment*).tw. (3212)

70 (SROI or ROI).tw. (21379)

71 or/69-70 (24017)

72 68 or 71 (1767789)

73 49 and 72 (47611)

74 exp United Kingdom/ (432881)

75 (national health service* or nhs*).ti,ab,in,ad. (393647)

76 (english not ((published or publication* or translat* or written or language* or speak* or literature or citation*) adj5 english)).ti,ab. (48979)

77 (gb or "g.b." or britain* or (british* not "british columbia") or uk or "u.k." or united kingdom* or (england* not "new england") or northern ireland* or northern irish* or scotland* or scottish* or ((wales or "south wales") not "new south wales") or welsh*).ti,ab,jx,in,ad. (3363228)

78 (bath or "bath's" or ((birmingham not alabama*) or ("birmingham's" not alabama*) or bradford or "bradford's" or brighton or "brighton's" or bristol or "bristol's" or carlisle* or "carlisle's" or (cambridge not (massachusetts* or boston* or harvard*)) or ("cambridge's" not (massachusetts* or boston* or harvard*)) or (canterbury not zealand*) or ("canterbury's" not zealand*) or chelmsford or "chelmsford's" or chester or "chester's" or chichester or "chichester's" or coventry or "coventry's" or derby or "derby's" or (durham not (carolina* or nc)) or ("durham's" not (carolina* or nc)) or ely or "ely's" or exeter or "exeter's" or gloucester or "gloucester's" or hereford or "hereford's" or hull or "hull's" or lancaster or "lancaster's" or leeds* or leicester or "leicester's" or (lincoln not nebraska*) or ("lincoln's" not nebraska*) or (liverpool not (new south wales* or nsw)) or ("liverpool's" not (new south wales* or nsw)) or ((london not (ontario* or ont or toronto*)) or ("london's" not (ontario* or ont or toronto*)) or manchester or "manchester's" or (newcastle not (new south wales* or nsw)) or ("newcastle's" not (new south wales* or nsw)) or norwich or "norwich's" or nottingham or "nottingham's" or oxford or "oxford's" or peterborough or "peterborough's" or plymouth or "plymouth's" or portsmouth or "portsmouth's" or preston or "preston's" or ripon or "ripon's" or salford or "salford's" or salisbury or "salisbury's" or sheffield or "sheffield's" or southampton or "southampton's" or st albans or stoke or "stoke's" or sunderland or "sunderland's" or truro or "truro's" or wakefield or "wakefield's" or wells or westminster or "westminster's" or winchester or "winchester's" or wolverhampton or "wolverhampton's" or (worcester not (massachusetts* or boston* or harvard*)) or ("worcester's" not (massachusetts* or boston* or harvard*)) or (york not ("new york*" or ny or ontario* or ont or toronto*)) or ("york's" not ("new york*" or ny or ontario* or ont or toronto*))))).ti,ab,in,ad. (2607993)

79 (bangor or "bangor's" or cardiff or "cardiff's" or newport or "newport's" or st asaph or "st asaph's" or st davids or swansea or "swansea's").ti,ab,in,ad. (106822)

80 (aberdeen or "aberdeen's" or dundee or "dundee's" or edinburgh or "edinburgh's" or glasgow or "glasgow's" or inverness or (perth not australia*) or ("perth's" not australia*) or stirling or "stirling's").ti,ab,in,ad. (358831)

81 (armagh or "armagh's" or belfast or "belfast's" or lisburn or "lisburn's" or londonderry or "londonderry's" or derry or "derry's" or newry or "newry's").ti,ab,in,ad. (48980)

82 or/74-81 (4103932)

83 (exp "arctic and antarctic"/ or exp oceanic regions/ or exp western hemisphere/ or exp africa/ or exp asia/) not (exp united kingdom/ or europe/) (3134377)

84 82 not 83 (3884792)

85 73 and 84 (5751)

86 animal/ (1522625)

87 exp animal experiment/ (2728526)

88 nonhuman/ (6628021)

89 (rat or rats or mouse or mice or hamster or hamsters or animal or animals or dog or dogs or cat or cats or bovine or sheep).ti,ab,sh. (5970209)

90 or/86-89 (9408162)

91 exp human/ (22604851)

92 human experiment/ (551499)

93 91 or 92 (22606761)

94 90 not (90 and 93) (6768070)

95 85 not 94 (5711)

96 limit 95 to yr="2000-Current" (5244)

97 conference.pt. (4926668)

98 96 not 97 (4195)

99 remove duplicates from 98 (4120)

**Key:**

/ or sh  = indexing term (Emtree Subject Heading)

exp = exploded indexing term (Emtree)

? = replaces 0 or 1 character

* = truncation

ti,ab,kw,tw = terms in either title, abstract, keyword or textword fields

jx,in,ad = terms in either journal word, institution, or correspondence address fields

adj3 = terms within three words of each other (any order)

**Econlit <1886 to August 05, 2021>**

via Ovid http://ovidsp.ovid.com/

Date range searched: 1886 to 5^th^ August 2021

Date searched: 16^th^ August 2021

Records retrieved: 428

The Econlit strategy below includes the NICE UK search filter for Ovid Medline (lines 26-32), which was adapted for use on this database.

Ayiku L, Levay P, Hudson T, Craven J, Barrett E, Finnegan A and Adams R. The MEDLINE UK filter: development and validation of a geographic search filter to retrieve research about the UK from OVID MEDLINE. Health Information and Libraries Journal, 2017 34 (3): 200-216. (Publisher: Wiley. © 2017 Crown copyright. Health Information and Libraries Journal © 2017 Health Libraries Group.)

1 (p?ediatric* or child* or preemie* or baby or babies or infant* or toddler* or neo nat* or neo-nat* or neonat* or newborn* or new-born* or newly born* or newly-born* or preschool* or pre-school* or schoolchild* or school-child* or schoolboy* or school-boy* or schoolgirl* or school-girl* or school-age* or prekindergarten or pre-kindergarten or kindergarten or boy* or girl* or kid* or LBW or VLBW or ELBW or "low birth weight").ti,ab. (35829)

2 (under adj (five* or "5") adj2 (age* or old*)).ti,ab. (19)

3 ("birth to 5" or "birth to five").ti,ab. (5)

4 or/1-3 (35834)

5 ((early or early-years or early-life*) adj3 (program* or interven* or scheme* or initiative*)).ti,ab. (744)

6 ((health* or wellness or welfare or well-being or wellbeing or safety or immuni*) adj5 (program* or interven* or scheme* or initiative* or encourag* or promot* or educat* or literacy or campaign* or improve* or improving)).ti,ab. (21070)

7 ((lifestyle* or diet* or food* or nutrition*) adj3 (intervention* or program* or chang* or modif* or improv* or enhanc* or adapt* or target* or alter* or impact*)).ti,ab. (4883)

8 ((behavio?r* or positive* or success*) adj3 (intervention* or program* or chang* or modif* or improv* or enhanc* or adapt* or impact*)).ti,ab. (20752)

9 ("best start in life" or "healthy child programme" or "healthy start programme" or "change for children").ti,ab. (10)

10 ((increas* or improv* or encourag* or support* or promot* or influen* or recommend* or motivat* or incentiv* or market* or advert* or subsid* or reward* or persua* or convinc* or instigat* or invest or benefit* or uptak* or start*) adj4 ((active or physically-active or health*) adj3 (living or life*))).ti,ab. (215)

11 ((increas* or improv* or encourag* or support* or promot* or influen* or recommend* or motivat* or incentiv* or market* or advert* or subsid* or reward* or persua* or convinc* or instigat* or invest or benefit* or uptak* or start*) adj4 (exercise* or exercising or fitness)).ti,ab. (1394)

12 ((decreas* or minimis* or reduc* or discourag* or disincentiv* or dissuade* or deter* or prevent* or avert* or divert) adj4 (physical* adj2 inactiv*)).ti,ab. (0)

13 ((decreas* or minimis* or reduc* or discourag* or disincentiv* or dissuade* or deter* or prevent* or avert* or divert) adj4 ((inactiv* or unhealthy) adj3 (living or life*))).ti,ab. (2)

14 ((increas* or improv* or encourag* or support* or promot* or influen* or recommend* or motivat* or incentiv* or market* or advert* or subsid* or reward* or persua* or convinc* or instigat* or invest or benefit* or uptak* or start*) adj4 ((weight or body mass or BMI) adj2 (healthy or manage* or control* or loss* or loos* or decreas* or reduc*))).ti,ab. (84)

15 ((decreas* or minimis* or reduc* or discourag* or disincentiv* or dissuade* or deter* or prevent* or avert* or divert) adj4 (obese or obesity or overweight)).ti,ab. (299)

16 ((increas* or improv* or encourag* or support* or promot* or influen* or recommend* or motivat* or incentiv* or market* or advert* or subsid* or reward* or persua* or convinc* or instigat* or invest or benefit* or uptak* or start*) adj4 ((health* or balanced) adj2 (diet* or eating or food or nutrition*))).ti,ab. (318)

17 ((increas* or improv* or encourag* or support* or promot* or influen* or recommend* or motivat* or incentiv* or market* or advert* or subsid* or reward* or persua* or convinc* or instigat* or invest or benefit* or uptak* or start*) adj4 ((salt or sugar or calorie*) adj2 (less or lessen or reduc* or restrict*))).ti,ab. (11)

18 ((decreas* or minimis* or reduc* or discourag* or disincentiv* or dissuade* or deter* or prevent* or avert* or divert) adj4 ((fizzy or sugary or sweetened) adj2 (drink or beverage*))).ti,ab. (12)

19 ((breastfeed* or feeding) adj3 (advice or advis* or educat* or support*)).ti,ab. (30)

20 ((decreas* or minimis* or reduc* or discourag* or disincentiv* or dissuade* or deter* or prevent* or avert* or divert) adj4 (malnutrition or malnourish* or undernourish* or overnutrition)).ti,ab. (132)

21 ((increas* or improv* or encourag* or support* or promot* or influen* or recommend* or motivat* or incentiv* or market* or advert* or subsid* or reward* or persua* or convinc* or instigat* or invest or benefit* or uptak* or start*) adj4 ((oral* or dental*) adj2 (health* or care or hygien*))).ti,ab. (29)

22 ((decreas* or minimis* or reduc* or discourag* or disincentiv* or dissuade* or deter* or prevent* or avert* or divert) adj4 ((oral* or dental* or tooth or teeth) adj2 (decay* or disease*))).ti,ab. (2)

23 ((identif* or detect* or prevent*) adj3 ((domestic* or spousal or child* or caregiver* or care-giver* or parent* or maternal* or paternal* or physical* or emotional*) adj2 (neglect* or abuse* or abusive or violen* or harm or maltreat* or mistreat*))).ti,ab. (16)

24 or/5-23 (47239)

25 4 and 24 (5029)

26 (national health service* or nhs*).ti,ab,kw,in. (962)

27 (gb or "g.b." or britain* or (british* not "british columbia") or uk or "u.k." or united kingdom* or (england* not "new england") or northern ireland* or northern irish* or scotland* or scottish* or ((wales or "south wales") not "new south wales") or welsh*).ti,ab,jx,in. (61046)

28 (bath or "bath's" or ((birmingham not alabama*) or ("birmingham's" not alabama*) or bradford or "bradford's" or brighton or "brighton's" or bristol or "bristol's" or carlisle* or "carlisle's" or (cambridge not (massachusetts* or boston* or harvard*)) or ("cambridge's" not (massachusetts* or boston* or harvard*)) or (canterbury not zealand*) or ("canterbury's" not zealand*) or chelmsford or "chelmsford's" or chester or "chester's" or chichester or "chichester's" or coventry or "coventry's" or derby or "derby's" or (durham not (carolina* or nc)) or ("durham's" not (carolina* or nc)) or ely or "ely's" or exeter or "exeter's" or gloucester or "gloucester's" or hereford or "hereford's" or hull or "hull's" or lancaster or "lancaster's" or leeds* or leicester or "leicester's" or (lincoln not nebraska*) or ("lincoln's" not nebraska*) or (liverpool not (new south wales* or nsw)) or ("liverpool's" not (new south wales* or nsw)) or ((london not (ontario* or ont or toronto*)) or ("london's" not (ontario* or ont or toronto*)) or manchester or "manchester's" or (newcastle not (new south wales* or nsw)) or ("newcastle's" not (new south wales* or nsw)) or norwich or "norwich's" or nottingham or "nottingham's" or oxford or "oxford's" or peterborough or "peterborough's" or plymouth or "plymouth's" or portsmouth or "portsmouth's" or preston or "preston's" or ripon or "ripon's" or salford or "salford's" or salisbury or "salisbury's" or sheffield or "sheffield's" or southampton or "southampton's" or st albans or stoke or "stoke's" or sunderland or "sunderland's" or truro or "truro's" or wakefield or "wakefield's" or wells or westminster or "westminster's" or winchester or "winchester's" or wolverhampton or "wolverhampton's" or (worcester not (massachusetts* or boston* or harvard*)) or ("worcester's" not (massachusetts* or boston* or harvard*)) or (york not ("new york*" or ny or ontario* or ont or toronto*)) or ("york's" not ("new york*" or ny or ontario* or ont or toronto*))))).ti,ab,in. (108728)

29 (bangor or "bangor's" or cardiff or "cardiff's" or newport or "newport's" or st asaph or "st asaph's" or st davids or swansea or "swansea's").ti,ab,in. (4140)

30 (aberdeen or "aberdeen's" or dundee or "dundee's" or edinburgh or "edinburgh's" or glasgow or "glasgow's" or inverness or (perth not australia*) or ("perth's" not australia*) or stirling or "stirling's").ti,ab,in. (8618)

31 (armagh or "armagh's" or belfast or "belfast's" or lisburn or "lisburn's" or londonderry or "londonderry's" or derry or "derry's" or newry or "newry's").ti,ab,in. (1412)

32 or/27-31 (159238)

33 25 and 32 (457)

34 limit 33 to yr="2000-Current" (428)

35 remove duplicates from 34 (428)

**Key:**

? = replaces 0 or 1 character

* = truncation

ti,ab  = terms in either title or abstract fields

jx,in = terms in either journal word or institution fields

adj3 = terms within three words of each other (any order)

**HMIC Health Management Information Consortium <1979 to July 2021>**

via Ovid http://ovidsp.ovid.com/

Date range searched: 1979 to July 2021

Date searched: 16^th^ August 2021

Records retrieved: 26

The HMIC strategy below includes the CADTH economics search filter for Ovid Medline (lines 44-60), which was adapted for use on this database.

Economic Evaluations/Cost/Economic Models - Ovid Medline. Strings attached: CADTH database search filters [Internet]. Ottawa: CADTH; 2016. [Accessed: 16 August 2021].

Available from: https://www.cadth.ca/resources/finding-evidence/strings-attached-cadths-database-search-filters#health

1 exp Paediatrics/ (625)

2 exp Pre School Children/ (539)

3 exp Infants/ (1821)

4 Toddlers/ (42)

5 (p?ediatric* or child* or preemie* or baby or babies or infant* or toddler* or neo nat* or neo-nat* or neonat* or newborn* or new-born* or newly born* or newly-born* or preschool* or pre-school* or schoolchild* or school-child* or schoolboy* or school-boy* or schoolgirl* or school-girl* or school-age* or prekindergarten or pre-kindergarten or kindergarten or boy* or girl* or kid* or LBW or VLBW or ELBW or "low birth weight").ti,ab. (37872)

6 (under adj (five* or "5") adj2 (age* or old*)).ti,ab. (61)

7 ("birth to 5" or "birth to five").ti,ab. (33)

8 or/1-7 (38312)

9 Public Health/ (11326)

10 Health Promotion/ (6745)

11 Health Literacy/ (210)

12 Health Education/ (2986)

13 Child Health/ (562)

14 Child Development/ (354)

15 Child Guidance/ (15)

16 Child Welfare/ (220)

17 Child Abuse/ (2148)

18 Infant Care/ (82)

19 exp Family Relations/ (544)

20 Physical Fitness/ (178)

21 Nutrition/ (1885)

22 Oral Health/ (412)

23 ((early or early-years or early-life*) adj3 (program* or interven* or scheme* or initiative*)).ti,ab. (991)

24 ((health* or wellness or welfare or well-being or wellbeing or safety or immuni*) adj5 (program* or interven* or scheme* or initiative* or encourag* or promot* or educat* or literacy or campaign* or improve* or improving)).ti,ab. (30548)

25 ((lifestyle* or diet* or food* or nutrition*) adj3 (intervention* or program* or chang* or modif* or improv* or enhanc* or adapt* or target* or alter* or impact*)).ti,ab. (2072)

26 ((behavio?r* or positive* or success*) adj3 (intervention* or program* or chang* or modif* or improv* or enhanc* or adapt* or impact*)).ti,ab. (5230)

27 ("best start in life" or "healthy child programme" or "healthy start programme" or "change for children").ti,ab. (124)

28 ((increas* or improv* or encourag* or support* or promot* or influen* or recommend* or motivat* or incentiv* or market* or advert* or subsid* or reward* or persua* or convinc* or instigat* or invest or benefit* or uptak* or start*) adj4 ((active or physically-active or health*) adj3 (living or life*))).ti,ab. (559)

29 ((increas* or improv* or encourag* or support* or promot* or influen* or recommend* or motivat* or incentiv* or market* or advert* or subsid* or reward* or persua* or convinc* or instigat* or invest or benefit* or uptak* or start*) adj4 (exercise* or exercising or fitness)).ti,ab. (663)

30 ((decreas* or minimis* or reduc* or discourag* or disincentiv* or dissuade* or deter* or prevent* or avert* or divert) adj4 (physical* adj2 inactiv*)).ti,ab. (19)

31 ((decreas* or minimis* or reduc* or discourag* or disincentiv* or dissuade* or deter* or prevent* or avert* or divert) adj4 ((inactiv* or unhealthy) adj3 (living or life*))).ti,ab. (7)

32 ((increas* or improv* or encourag* or support* or promot* or influen* or recommend* or motivat* or incentiv* or market* or advert* or subsid* or reward* or persua* or convinc* or instigat* or invest or benefit* or uptak* or start*) adj4 ((weight or body mass or BMI) adj2 (healthy or manage* or control* or loss* or loos* or decreas* or reduc*))).ti,ab. (243)

33 ((decreas* or minimis* or reduc* or discourag* or disincentiv* or dissuade* or deter* or prevent* or avert* or divert) adj4 (obese or obesity or overweight)).ti,ab. (787)

34 ((increas* or improv* or encourag* or support* or promot* or influen* or recommend* or motivat* or incentiv* or market* or advert* or subsid* or reward* or persua* or convinc* or instigat* or invest or benefit* or uptak* or start*) adj4 ((health* or balanced) adj2 (diet* or eating or food or nutrition*))).ti,ab. (360)

35 ((increas* or improv* or encourag* or support* or promot* or influen* or recommend* or motivat* or incentiv* or market* or advert* or subsid* or reward* or persua* or convinc* or instigat* or invest or benefit* or uptak* or start*) adj4 ((salt or sugar or calorie*) adj2 (less or lessen or reduc* or restrict*))).ti,ab. (16)

36 ((decreas* or minimis* or reduc* or discourag* or disincentiv* or dissuade* or deter* or prevent* or avert* or divert) adj4 ((fizzy or sugary or sweetened) adj2 (drink or beverage*))).ti,ab. (13)

37 ((breastfeed* or feeding) adj3 (advice or advis* or educat* or support*)).ti,ab. (181)

38 ((decreas* or minimis* or reduc* or discourag* or disincentiv* or dissuade* or deter* or prevent* or avert* or divert) adj4 (malnutrition or malnourish* or undernourish* or overnutrition)).ti,ab. (33)

39 ((increas* or improv* or encourag* or support* or promot* or influen* or recommend* or motivat* or incentiv* or market* or advert* or subsid* or reward* or persua* or convinc* or instigat* or invest or benefit* or uptak* or start*) adj4 ((oral* or dental*) adj2 (health* or care or hygien*))).ti,ab. (310)

40 ((decreas* or minimis* or reduc* or discourag* or disincentiv* or dissuade* or deter* or prevent* or avert* or divert) adj4 ((oral* or dental* or tooth or teeth) adj2 (decay* or disease*))).ti,ab. (38)

41 ((identif* or detect* or prevent*) adj3 ((domestic* or spousal or child* or caregiver* or care-giver* or parent* or maternal* or paternal* or physical* or emotional*) adj2 (neglect* or abuse* or abusive or violen* or harm or maltreat* or mistreat*))).ti,ab. (209)

42 or/9-41 (53443)

43 8 and 42 (9507)

44 Economics/ (600)

45 exp "Cost Analysis"/ (340)

46 exp Health Economics/ (3701)

47 exp "Cost Effectiveness"/ (5694)

48 exp Economic Evaluation/ (1451)

49 budget*.ti,ab. (4839)

50 (economic* or cost or costs or costly or costing or price or prices or pricing or pharmacoeconomic* or pharmaco-economic* or expenditure or expenditures or expense or expenses or financial or finance or finances or financed).ti,ab. (48287)

51 (cost* adj2 (effective* or utilit* or benefit* or minimi* or analy* or outcome or outcomes)).ab. (7533)

52 (value adj2 (money or monetary)).ti,ab. (1189)

53 exp Economic Models/ (169)

54 economic model*.ab. (253)

55 markov.ti,ab. (251)

56 Monte Carlo Methods/ (5)

57 monte carlo.ti,ab. (118)

58 exp Decision Theory/ (18)

59 (decision* adj2 (tree* or analy* or model*)).ti,ab. (640)

60 or/44-59 (54793)

61 (return adj3 investment*).ti,ab. (149)

62 (SROI or ROI).ti,ab. (36)

63 or/61-62 (169)

64 60 or 63 (54847)

65 43 and 64 (1188)

66 limit 65 to yr="2000-Current" (26)

67 remove duplicates from 66 (26)

**Key:**

/ = indexing term (Subject Heading)

exp = exploded indexing term (Emtree)

? = replaces 0 or 1 character

* = truncation

ti,ab  = terms in either title or abstract fields

adj3 = terms within three words of each other (any order)

**Cochrane Central Register of Controlled Trials (CENTRAL)**

via Wiley http://onlinelibrary.wiley.com/

Date range searched: Issue 8 of 12, August 2021

Date searched: 23^rd^ August 2021

Records retrieved: 4029

The CENTRAL strategy below includes part of the CADTH economics search filter for Ovid Medline (lines 52-73) and part of the NICE UK search filter for Ovid Medline (lines 81-82), both of which were adapted for use on this database.

Economic Evaluations/Cost/Economic Models - Ovid Medline. Strings attached: CADTH database search filters [Internet]. Ottawa: CADTH; 2016. [Accessed: 16 August 2021].

Available from: https://www.cadth.ca/resources/finding-evidence/strings-attached-cadths-database-search-filters#health

Ayiku L, Levay P, Hudson T, Craven J, Barrett E, Finnegan A and Adams R. The MEDLINE UK filter: development and validation of a geographic search filter to retrieve research about the UK from OVID MEDLINE. Health Information and Libraries Journal, 2017 34 (3): 200-216. (Publisher: Wiley. © 2017 Crown copyright. Health Information and Libraries Journal © 2017 Health Libraries Group.)

#1 [mh Pediatrics] 706

#2 [mh Child] 58154

#3 [mh Infant] 33195

#4 [mh "Infant, Newborn"] 16573

#5 [mh "Infant, Low Birth Weight"] 2250

#6 [mh "Infant, Very Low Birth Weight"] 990

#7 [mh "Infant, Premature"] 3943

#8 (p*diatric* or child* or preemie* or baby or babies or infant* or toddler* or neo NEXT nat* or neonat* or newborn* or new NEXT born* or newly NEXT born* or preschool* or pre NEXT school* or schoolchild* or school NEXT child* or schoolboy* or school NEXT boy* or schoolgirl* or school NEXT girl* or school NEXT age* or prekindergarten or pre NEXT kindergarten or kindergarten or boy* or girl* or kid* or LBW or VLBW or ELBW or "low birth weight"):ti,ab 223044

#9 (under NEAR (five* or "5") NEAR/2 (age* or old*)):ti,ab 509

#10 ("birth to 5" or "birth to five"):ti,ab 32

#11 {OR #1-#10} 239541

#12 [mh ^"Public Health"] 260

#13 [mh ^"Health Promotion"] 6089

#14 [mh ^"Health Literacy"] 399

#15 [mh ^"Health Education"] 4049

#16 [mh ^"Social Determinants of Health"] 23

#17 [mh ^"Child Health"] 129

#18 [mh ^"Child Development"] 1985

#19 [mh ^"Child Guidance"] 10

#20 [mh ^"Child Welfare"] 333

#21 [mh ^"Child Abuse"] 370

#22 [mh ^"Child Nutrition Disorders"] 240

#23 [mh ^"Infant Health"] 56

#24 [mh ^"Infant Welfare"] 83

#25 [mh "Family Relations"] 3282

#26 [mh ^"Early Intervention, Educational"] 516

#27 [mh ^"Early Medical Intervention"] 414

#28 [mh "Physical Fitness"] 3503

#29 [mh ^"Diet, Healthy"] 543

#30 [mh ^"Oral Health"] 451

#31 ((early or early NEXT years or early NEXT life*) NEAR/3 (program* or interven* or scheme* or initiative*)):ti,ab 7685

#32 ((health* or wellness or welfare or well NEXT being or wellbeing or safety or immuni*) NEAR/5 (program* or interven* or scheme* or initiative* or encourag* or promot* or educat* or literacy or campaign* or improve* or improving)):ti,ab 70290

#33 ((lifestyle* or diet* or food* or nutrition*) NEAR/3 (intervention* or program* or chang* or modif* or improv* or enhanc* or adapt* or target* or alter* or impact*)):ti,ab 37420

#34 ((behavi*r* or positive* or success*) NEAR/3 (intervention* or program* or chang* or modif* or improv* or enhanc* or adapt* or impact*)):ti,ab 49659

#35 ("best start in life" or "healthy child programme" or "healthy start programme" or "change for children"):ti,ab 30

#36 ((increas* or improv* or encourag* or support* or promot* or influen* or recommend* or motivat* or incentiv* or market* or advert* or subsid* or reward* or persua* or convinc* or instigat* or invest or benefit* or uptak* or start*) NEAR/4 ((active or physically NEXT active or health*) NEAR/3 (living or life*))):ti,ab 4358

#37 ((increas* or improv* or encourag* or support* or promot* or influen* or recommend* or motivat* or incentiv* or market* or advert* or subsid* or reward* or persua* or convinc* or instigat* or invest or benefit* or uptak* or start*) NEAR/4 (exercise* or exercising or fitness)):ti,ab 28608

#38 ((decreas* or minimis* or reduc* or discourag* or disincentiv* or dissuade* or deter* or prevent* or avert* or divert) NEAR/4 (physical* NEAR/2 inactiv*)):ti,ab 79

#39 ((decreas* or minimis* or reduc* or discourag* or disincentiv* or dissuade* or deter* or prevent* or avert* or divert) NEAR/4 ((inactiv* or unhealthy) NEAR/3 (living or life*))):ti,ab 13

#40 ((increas* or improv* or encourag* or support* or promot* or influen* or recommend* or motivat* or incentiv* or market* or advert* or subsid* or reward* or persua* or convinc* or instigat* or invest or benefit* or uptak* or start*) NEAR/4 ((weight or body NEXT mass or BMI) NEAR/2 (healthy or manage* or control* or loss* or loos* or decreas* or reduc*))):ti,ab 6561

#41 ((decreas* or minimis* or reduc* or discourag* or disincentiv* or dissuade* or deter* or prevent* or avert* or divert) NEAR/4 (obese or obesity or overweight)):ti,ab 5356

#42 ((increas* or improv* or encourag* or support* or promot* or influen* or recommend* or motivat* or incentiv* or market* or advert* or subsid* or reward* or persua* or convinc* or instigat* or invest or benefit* or uptak* or start*) NEAR/4 ((health* or balanced) NEAR/2 (diet* or eating or food or nutrition*))):ti,ab 1974

#43 ((increas* or improv* or encourag* or support* or promot* or influen* or recommend* or motivat* or incentiv* or market* or advert* or subsid* or reward* or persua* or convinc* or instigat* or invest or benefit* or uptak* or start*) NEAR/4 ((salt or sugar or calorie*) NEAR/2 (less or lessen or reduc* or restrict*))):ti,ab 362

#44 ((decreas* or minimis* or reduc* or discourag* or disincentiv* or dissuade* or deter* or prevent* or avert* or divert) NEAR/4 ((fizzy or sugary or sweetened) NEAR/2 (drink or beverage*))):ti,ab 160

#45 ((breastfeed* or feeding) NEAR/3 (advice or advis* or educat* or support*)):ti,ab 947

#46 ((decreas* or minimis* or reduc* or discourag* or disincentiv* or dissuade* or deter* or prevent* or avert* or divert) NEAR/4 (malnutrition or malnourish* or undernourish* or overnutrition)):ti,ab 473

#47 ((increas* or improv* or encourag* or support* or promot* or influen* or recommend* or motivat* or incentiv* or market* or advert* or subsid* or reward* or persua* or convinc* or instigat* or invest or benefit* or uptak* or start*) NEAR/4 ((oral* or dental*) NEAR/2 (health* or care or hygien*))):ti,ab 1515

#48 ((decreas* or minimis* or reduc* or discourag* or disincentiv* or dissuade* or deter* or prevent* or avert* or divert) NEAR/4 ((oral* or dental* or tooth or teeth) NEAR/2 (decay* or disease*))):ti,ab 215

#49 ((identif* or detect* or prevent*) NEAR/3 ((domestic* or spousal or child* or caregiver* or care NEXT giver* or parent* or maternal* or paternal* or physical* or emotional*) NEAR/2 (neglect* or abuse* or abusive or violen* or harm or maltreat* or mistreat*))):ti,ab 261

#50 {OR #12-#49} 179460

#51 (#11 and #50) 38754

#52 [mh ^Economics] 41

#53 [mh "Costs and Cost Analysis"] 10920

#54 [mh ^"Economics, Nursing"] 12

#55 [mh ^"Economics, Medical"] 26

#56 [mh ^"Economics, Pharmaceutical"] 65

#57 [mh "Economics, Hospital"] 728

#58 [mh ^"Economics, Dental"] 2

#59 [mh "Fees and Charges"] 258

#60 [mh Budgets] 28

#61 (budget*):ti,ab,kw 1217

#62 (economic* or cost or costs or costly or costing or price or prices or pricing or pharmacoeconomic* or expenditure? or expense? or financial or finance?):ti,ab,kw 97296

#63 (cost* NEAR/2 (effective* or utilit* or benefit* or minimi* or analy* or outcome?)):ab,kw 35189

#64 (value NEAR/2 (money or monetary)):ti,ab,kw 336

#65 [mh "Models, Economic"] 362

#66 (economic NEXT model*):ab,kw 329

#67 [mh ^"Markov Chains"] 278

#68 (markov):ti,ab,kw 1469

#69 [mh ^"Monte Carlo Method"] 192

#70 (monte carlo):ti,ab,kw 916

#71 [mh "Decision Theory"] 168

#72 (decision* NEAR/2 (tree* or analy* or model*)):ti,ab,kw 2398

#73 {OR #52-#72} 99711

#74 (return NEAR/3 investment*):ti,ab 156

#75 (SROI or ROI):ti,ab 691

#76 {OR #74-#75} 815

#77 (#73 or #76) 100355

#78 (#51 and #77) 5402

#79 (rat or rats or rodent* or mouse or mice or "mus musculus" or "mus domesticus" or murine or murinae or bovine or sheep or ovine or "ovis aries" or porcine):ti,ab,kw 14911

#80 #78 not #79 5361

#81 [mh Africa] 7534

#82 [mh Americas] 26750

#83 [mh "Antarctic Regions"] 12

#84 [mh "Arctic Regions"] 7

#85 [mh Asia] 21466

#86 [mh Australia] 4512

#87 [mh Oceania] 5181

#88 {OR #81-#87} 59504

#89 [mh "United Kingdom"] 6598

#90 [mh ^Europe] 2478

#91 {OR #89-#90} 9062

#92 #88 not #91 58222

#93 #80 not #92 with Publication Year from 2000 to 2021, in Trials 4029

**Key:**

mh = indexing term, exploded (MeSH)

mh ^ = indexing term, unexploded (MeSH)

* = truncation

? = 1 additional character

ti,ab,kw = terms in either title or abstract or keyword fields

near/3 = terms within three words of each other (any order)

next = terms are next to each other

**Cochrane Database of Systematic Reviews (CDSR)**

via Wiley <http://onlinelibrary.wiley.com/>

Date range searched: Issue 8 of 12, August 2021

Date searched: 16^th^ August 2021

Records retrieved: 241

The CDSR strategy below includes part of the CADTH economics search filter for Ovid Medline (lines 52-73) and part of the NICE UK search filter for Ovid Medline (lines 81-82), both of which were adapted by the Information Specialist for use on this database.

Economic Evaluations/Cost/Economic Models - Ovid Medline. Strings attached: CADTH database search filters [Internet]. Ottawa: CADTH; 2016. [Accessed: 16 August 2021].

Available from: https://www.cadth.ca/resources/finding-evidence/strings-attached-cadths-database-search-filters#health

Ayiku L, Levay P, Hudson T, Craven J, Barrett E, Finnegan A and Adams R. The MEDLINE UK filter: development and validation of a geographic search filter to retrieve research about the UK from OVID MEDLINE. Health Information and Libraries Journal, 2017 34 (3): 200-216. (Publisher: Wiley. © 2017 Crown copyright. Health Information and Libraries Journal © 2017 Health Libraries Group.)

#1 [mh Pediatrics] 706

#2 [mh Child] 58154

#3 [mh Infant] 33195

#4 [mh "Infant, Newborn"] 16573

#5 [mh "Infant, Low Birth Weight"] 2250

#6 [mh "Infant, Very Low Birth Weight"] 990

#7 [mh "Infant, Premature"] 3943

#8 (p*diatric* or child* or preemie* or baby or babies or infant* or toddler* or neo NEXT nat* or neonat* or newborn* or new NEXT born* or newly NEXT born* or preschool* or pre NEXT school* or schoolchild* or school NEXT child* or schoolboy* or school NEXT boy* or schoolgirl* or school NEXT girl* or school NEXT age* or prekindergarten or pre NEXT kindergarten or kindergarten or boy* or girl* or kid* or LBW or VLBW or ELBW or "low birth weight"):ti,ab 223044

#9 (under NEAR (five* or "5") NEAR/2 (age* or old*)):ti,ab 509

#10 ("birth to 5" or "birth to five"):ti,ab 32

#11 {OR #1-#10} 239541

#12 [mh ^"Public Health"] 260

#13 [mh ^"Health Promotion"] 6089

#14 [mh ^"Health Literacy"] 399

#15 [mh ^"Health Education"] 4049

#16 [mh ^"Social Determinants of Health"] 23

#17 [mh ^"Child Health"] 129

#18 [mh ^"Child Development"] 1985

#19 [mh ^"Child Guidance"] 10

#20 [mh ^"Child Welfare"] 333

#21 [mh ^"Child Abuse"] 370

#22 [mh ^"Child Nutrition Disorders"] 240

#23 [mh ^"Infant Health"] 56

#24 [mh ^"Infant Welfare"] 83

#25 [mh "Family Relations"] 3282

#26 [mh ^"Early Intervention, Educational"] 516

#27 [mh ^"Early Medical Intervention"] 414

#28 [mh "Physical Fitness"] 3503

#29 [mh ^"Diet, Healthy"] 543

#30 [mh ^"Oral Health"] 451

#31 ((early or early NEXT years or early NEXT life*) NEAR/3 (program* or interven* or scheme* or initiative*)):ti,ab 7685

#32 ((health* or wellness or welfare or well NEXT being or wellbeing or safety or immuni*) NEAR/5 (program* or interven* or scheme* or initiative* or encourag* or promot* or educat* or literacy or campaign* or improve* or improving)):ti,ab 70290

#33 ((lifestyle* or diet* or food* or nutrition*) NEAR/3 (intervention* or program* or chang* or modif* or improv* or enhanc* or adapt* or target* or alter* or impact*)):ti,ab 37420

#34 ((behavi*r* or positive* or success*) NEAR/3 (intervention* or program* or chang* or modif* or improv* or enhanc* or adapt* or impact*)):ti,ab 49659

#35 ("best start in life" or "healthy child programme" or "healthy start programme" or "change for children"):ti,ab 30

#36 ((increas* or improv* or encourag* or support* or promot* or influen* or recommend* or motivat* or incentiv* or market* or advert* or subsid* or reward* or persua* or convinc* or instigat* or invest or benefit* or uptak* or start*) NEAR/4 ((active or physically NEXT active or health*) NEAR/3 (living or life*))):ti,ab 4358

#37 ((increas* or improv* or encourag* or support* or promot* or influen* or recommend* or motivat* or incentiv* or market* or advert* or subsid* or reward* or persua* or convinc* or instigat* or invest or benefit* or uptak* or start*) NEAR/4 (exercise* or exercising or fitness)):ti,ab 28608

#38 ((decreas* or minimis* or reduc* or discourag* or disincentiv* or dissuade* or deter* or prevent* or avert* or divert) NEAR/4 (physical* NEAR/2 inactiv*)):ti,ab 79

#39 ((decreas* or minimis* or reduc* or discourag* or disincentiv* or dissuade* or deter* or prevent* or avert* or divert) NEAR/4 ((inactiv* or unhealthy) NEAR/3 (living or life*))):ti,ab 13

#40 ((increas* or improv* or encourag* or support* or promot* or influen* or recommend* or motivat* or incentiv* or market* or advert* or subsid* or reward* or persua* or convinc* or instigat* or invest or benefit* or uptak* or start*) NEAR/4 ((weight or body NEXT mass or BMI) NEAR/2 (healthy or manage* or control* or loss* or loos* or decreas* or reduc*))):ti,ab 6561

#41 ((decreas* or minimis* or reduc* or discourag* or disincentiv* or dissuade* or deter* or prevent* or avert* or divert) NEAR/4 (obese or obesity or overweight)):ti,ab 5356

#42 ((increas* or improv* or encourag* or support* or promot* or influen* or recommend* or motivat* or incentiv* or market* or advert* or subsid* or reward* or persua* or convinc* or instigat* or invest or benefit* or uptak* or start*) NEAR/4 ((health* or balanced) NEAR/2 (diet* or eating or food or nutrition*))):ti,ab 1974

#43 ((increas* or improv* or encourag* or support* or promot* or influen* or recommend* or motivat* or incentiv* or market* or advert* or subsid* or reward* or persua* or convinc* or instigat* or invest or benefit* or uptak* or start*) NEAR/4 ((salt or sugar or calorie*) NEAR/2 (less or lessen or reduc* or restrict*))):ti,ab 362

#44 ((decreas* or minimis* or reduc* or discourag* or disincentiv* or dissuade* or deter* or prevent* or avert* or divert) NEAR/4 ((fizzy or sugary or sweetened) NEAR/2 (drink or beverage*))):ti,ab 160

#45 ((breastfeed* or feeding) NEAR/3 (advice or advis* or educat* or support*)):ti,ab 947

#46 ((decreas* or minimis* or reduc* or discourag* or disincentiv* or dissuade* or deter* or prevent* or avert* or divert) NEAR/4 (malnutrition or malnourish* or undernourish* or overnutrition)):ti,ab 473

#47 ((increas* or improv* or encourag* or support* or promot* or influen* or recommend* or motivat* or incentiv* or market* or advert* or subsid* or reward* or persua* or convinc* or instigat* or invest or benefit* or uptak* or start*) NEAR/4 ((oral* or dental*) NEAR/2 (health* or care or hygien*))):ti,ab 1515

#48 ((decreas* or minimis* or reduc* or discourag* or disincentiv* or dissuade* or deter* or prevent* or avert* or divert) NEAR/4 ((oral* or dental* or tooth or teeth) NEAR/2 (decay* or disease*))):ti,ab 215

#49 ((identif* or detect* or prevent*) NEAR/3 ((domestic* or spousal or child* or caregiver* or care NEXT giver* or parent* or maternal* or paternal* or physical* or emotional*) NEAR/2 (neglect* or abuse* or abusive or violen* or harm or maltreat* or mistreat*))):ti,ab 261

#50 {OR #12-#49} 179460

#51 (#11 and #50) 38754

#52 [mh ^Economics] 41

#53 [mh "Costs and Cost Analysis"] 10920

#54 [mh ^"Economics, Nursing"] 12

#55 [mh ^"Economics, Medical"] 26

#56 [mh ^"Economics, Pharmaceutical"] 65

#57 [mh "Economics, Hospital"] 728

#58 [mh ^"Economics, Dental"] 2

#59 [mh "Fees and Charges"] 258

#60 [mh Budgets] 28

#61 (budget*):ti,ab,kw 1217

#62 (economic* or cost or costs or costly or costing or price or prices or pricing or pharmacoeconomic* or expenditure? or expense? or financial or finance?):ti,ab,kw 97296

#63 (cost* NEAR/2 (effective* or utilit* or benefit* or minimi* or analy* or outcome?)):ab,kw 35189

#64 (value NEAR/2 (money or monetary)):ti,ab,kw 336

#65 [mh "Models, Economic"] 362

#66 (economic NEXT model*):ab,kw 329

#67 [mh ^"Markov Chains"] 278

#68 (markov):ti,ab,kw 1469

#69 [mh ^"Monte Carlo Method"] 192

#70 (monte carlo):ti,ab,kw 916

#71 [mh "Decision Theory"] 168

#72 (decision* NEAR/2 (tree* or analy* or model*)):ti,ab,kw 2398

#73 {OR #52-#72} 99711

#74 (return NEAR/3 investment*):ti,ab 156

#75 (SROI or ROI):ti,ab 691

#76 {OR #74-#75} 815

#77 (#73 or #76) 100355

#78 (#51 and #77) 5402

#79 (rat or rats or rodent* or mouse or mice or "mus musculus" or "mus domesticus" or murine or murinae or bovine or sheep or ovine or "ovis aries" or porcine):ti,ab,kw 14911

#80 #78 not #79 5361

#81 [mh Africa] 7534

#82 [mh Americas] 26750

#83 [mh "Antarctic Regions"] 12

#84 [mh "Arctic Regions"] 7

#85 [mh Asia] 21466

#86 [mh Australia] 4512

#87 [mh Oceania] 5181

#88 {OR #81-#87} 59504

#89 [mh "United Kingdom"] 6598

#90 [mh ^Europe] 2478

#91 {OR #89-#90} 9062

#92 #88 not #91 58222

#93 #80 not #92 with Cochrane Library publication date Between Jan 2000 and Aug 2021, in Cochrane Reviews 241

**Key:**

mh = indexing term, exploded (MeSH)

mh ^ = indexing term, unexploded (MeSH)

* = truncation

? = 1 additional character

ti,ab,kw = terms in either title or abstract or keyword fields

near/3 = terms within three words of each other (any order)

next = terms are next to each other

**NHS EED**

via https://www.crd.york.ac.uk/CRDWeb/

Date range searched: Inception to 31^st^ March 2015.

Date searched: 16^th^ August 2021

Records retrieved: 811

1 MeSH DESCRIPTOR Pediatrics 112

2 MeSH DESCRIPTOR Child EXPLODE ALL TREES IN NHSEED 1680

3 MeSH DESCRIPTOR Infant EXPLODE ALL TREES IN NHSEED 1251

4 MeSH DESCRIPTOR Infant, Newborn EXPLODE ALL TREES IN NHSEED 685

5 MeSH DESCRIPTOR Infant, Low Birth Weight EXPLODE ALL TREES IN NHSEED 53

6 MeSH DESCRIPTOR Infant, Very Low Birth Weight EXPLODE ALL TREES IN NHSEED 22

7 MeSH DESCRIPTOR Infant, Premature EXPLODE ALL TREES IN NHSEED 64

8 (pediatric* or paediatric* or child* or preemie* or baby or babies or infant* or toddler* or neo nat* or neo-nat* or neonat* or newborn* or new-born* or newly born* or newly-born* or preschool* or pre-school* or schoolchild* or school-child* or schoolboy* or school-boy* or schoolgirl* or school-girl* or school-age* or prekindergarten or pre-kindergarten or kindergarten or boy* or girl* or kid* or LBW or VLBW or ELBW) IN NHSEED 3467

9 (under NEAR (five* or 5) NEAR2 (age* or old*)) IN NHSEED 29

10 (birth NEAR (5 or five)) IN NHSEED 33

11 (low NEAR (birth weight or birthweight)) IN NHSEED 74

12 #1 OR #2 OR #3 OR #4 OR #5 OR #6 OR #7 OR #8 OR #9 OR #10 OR #11 3544

13 MeSH DESCRIPTOR Public Health IN NHSEED 68

14 MeSH DESCRIPTOR Health Promotion IN NHSEED 226

15 MeSH DESCRIPTOR Health Literacy IN NHSEED 1

16 MeSH DESCRIPTOR Health Education IN NHSEED 84

17 MeSH DESCRIPTOR Social Determinants of Health IN NHSEED 0

18 MeSH DESCRIPTOR Child Health IN NHSEED 0

19 MeSH DESCRIPTOR Child Development IN NHSEED 9

20 MeSH DESCRIPTOR Child Guidance IN NHSEED 0

21 MeSH DESCRIPTOR Child Welfare IN NHSEED 20

22 MeSH DESCRIPTOR Child Abuse IN NHSEED 7

23 MeSH DESCRIPTOR Child Nutrition Disorders IN NHSEED 5

24 MeSH DESCRIPTOR Infant Health IN NHSEED 0

25 MeSH DESCRIPTOR Infant Welfare IN NHSEED 5

26 MeSH DESCRIPTOR Family Relations EXPLODE ALL TREES IN NHSEED 21

27 MeSH DESCRIPTOR Early Intervention, Educational IN NHSEED 0

28 MeSH DESCRIPTOR Early Medical Intervention IN NHSEED 18

29 MeSH DESCRIPTOR Physical Fitness EXPLODE ALL TREES IN NHSEED 16

30 MeSH DESCRIPTOR Diet, Healthy IN NHSEED 0

31 MeSH DESCRIPTOR Oral Health IN NHSEED 10

32 ((early or early-years or early-life*) NEAR3 (program* or interven* or scheme* or initiative*)) IN NHSEED 113

33 ((health* or wellness or welfare or well-being or wellbeing or safety or immuni*) NEAR5 (program* or interven* or scheme* or initiative* or encourag* or promot* or educat* or literacy or campaign* or improve* or improving)) IN NHSEED 2662

34 ((lifestyle* or diet* or food* or nutrition*) NEAR3 (intervention* or program* or chang* or modif* or improv* or enhanc* or adapt* or target* or alter* or impact*)) IN NHSEED 177

35 ((behavior* or behaviour* or positive* or success*) NEAR3 (intervention* or program* or chang* or modif* or improv* or enhanc* or adapt* or impact*)) IN NHSEED 297

36 ((increas* or improv* or encourag* or support* or promot* or influen* or recommend* or motivat* or incentiv* or market* or advert* or subsid* or reward* or persua* or convinc* or instigat* or invest or benefit* or uptak* or start*) NEAR4 ((active or physically-active or health*) NEAR3 (living or life*))) IN NHSEED 62

37 ((increas* or improv* or encourag* or support* or promot* or influen* or recommend* or motivat* or incentiv* or market* or advert* or subsid* or reward* or persua* or convinc* or instigat* or invest or benefit* or uptak* or start*) NEAR4 (exercise* or exercising or fitness)) IN NHSEED 165

38 ((decreas* or minimis* or reduc* or discourag* or disincentiv* or dissuade* or deter* or prevent* or avert* or divert) NEAR4 (physical* NEAR2 inactiv*)) IN NHSEED 0

39 ((decreas* or minimis* or reduc* or discourag* or disincentiv* or dissuade* or deter* or prevent* or avert* or divert) NEAR4 ((inactiv* or unhealthy) NEAR3 (living or life*))) IN NHSEED 0

40 ((increas* or improv* or encourag* or support* or promot* or influen* or recommend* or motivat* or incentiv* or market* or advert* or subsid* or reward* or persua* or convinc* or instigat* or invest or benefit* or uptak* or start*) NEAR4 ((weight or body mass or BMI) NEAR2 (healthy or manage* or control* or loss* or loos* or decreas* or reduc*))) IN NHSEED 11

41 ((decreas* or minimis* or reduc* or discourag* or disincentiv* or dissuade* or deter* or prevent* or avert* or divert) NEAR4 (obese or obesity or overweight)) IN NHSEED 44

42 ((increas* or improv* or encourag* or support* or promot* or influen* or recommend* or motivat* or incentiv* or market* or advert* or subsid* or reward* or persua* or convinc* or instigat* or invest or benefit* or uptak* or start*) NEAR4 ((health* or balanced) NEAR2 (diet* or eating or food or nutrition*))) IN NHSEED 4

43 ((increas* or improv* or encourag* or support* or promot* or influen* or recommend* or motivat* or incentiv* or market* or advert* or subsid* or reward* or persua* or convinc* or instigat* or invest or benefit* or uptak* or start*) NEAR4 ((salt or sugar or calorie*) NEAR2 (less or lessen or reduc* or restrict*))) IN NHSEED 0

44 ((decreas* or minimis* or reduc* or discourag* or disincentiv* or dissuade* or deter* or prevent* or avert* or divert) NEAR4 ((fizzy or sugary or sweetened) NEAR2 (drink or beverage*))) IN NHSEED 0

45 ((breastfeed* or feeding) NEAR3 (advice or advis* or educat* or support*)) IN NHSEED 4

46 ((decreas* or minimis* or reduc* or discourag* or disincentiv* or dissuade* or deter* or prevent* or avert* or divert) NEAR4 (malnutrition or malnourish* or undernourish* or overnutrition)) IN NHSEED 3

47 ((increas* or improv* or encourag* or support* or promot* or influen* or recommend* or motivat* or incentiv* or market* or advert* or subsid* or reward* or persua* or convinc* or instigat* or invest or benefit* or uptak* or start*) NEAR4 ((oral* or dental*) NEAR2 (health* or care or hygien*))) IN NHSEED 21

48 ((decreas* or minimis* or reduc* or discourag* or disincentiv* or dissuade* or deter* or prevent* or avert* or divert) NEAR4 ((oral* or dental* or tooth or teeth) NEAR2 (decay* or disease*))) IN NHSEED 3

49 ((identif* or detect* or prevent*) NEAR3 ((domestic* or spousal or child* or caregiver* or care-giver* or parent* or maternal* or paternal* or physical* or emotional*) NEAR2 (neglect* or abuse* or abusive or violen* or harm or maltreat* or mistreat*))) IN NHSEED 2

50 #13 OR #14 OR #15 OR #16 OR #17 OR #18 OR #19 OR #20 OR #21 OR #22 OR #23 OR #24 OR #25 OR #26 OR #27 OR #28 OR #29 OR #30 OR #31 OR #32 OR #33 OR #34 OR #35 OR #36 OR #37 OR #38 OR #39 OR #40 OR #41 OR #42 OR #43 OR #44 OR #45 OR #46 OR #47 OR #48 OR #49 3259

51 #12 AND #50 927

52 * IN NHSEED FROM 2000 TO 2015 14762

53 #51 AND #52 811

**Key:**

MeSH DESCRIPTOR = indexing term: Medical Subject Heading (MeSH)

EXPLODE ALL TREES = exploded indexing term (MeSH)

* = truncation

NEAR3 = terms within three words of each other (only in the order specified).

**HTA**

via https://www.crd.york.ac.uk/CRDWeb/

Date range searched: Inception to March 2018

Date searched: 16^th^ August 2021

Records retrieved: 447

1 MeSH DESCRIPTOR Pediatrics 112

2 MeSH DESCRIPTOR Child EXPLODE ALL TREES IN HTA 572

3 MeSH DESCRIPTOR Infant EXPLODE ALL TREES IN HTA 317

4 MeSH DESCRIPTOR Infant, Newborn EXPLODE ALL TREES IN HTA 205

5 MeSH DESCRIPTOR Infant, Low Birth Weight EXPLODE ALL TREES IN HTA 8

6 MeSH DESCRIPTOR Infant, Very Low Birth Weight EXPLODE ALL TREES IN HTA 4

7 MeSH DESCRIPTOR Infant, Premature EXPLODE ALL TREES IN HTA 25

8 (pediatric* or paediatric* or child* or preemie* or baby or babies or infant* or toddler* or neo nat* or neo-nat* or neonat* or newborn* or new-born* or newly born* or newly-born* or preschool* or pre-school* or schoolchild* or school-child* or schoolboy* or school-boy* or schoolgirl* or school-girl* or school-age* or prekindergarten or pre-kindergarten or kindergarten or boy* or girl* or kid* or LBW or VLBW or ELBW) IN HTA 1931

9 (under NEAR (five* or 5) NEAR2 (age* or old*)) IN HTA 4

10 (birth NEAR (5 or five)) IN HTA 1

11 (low NEAR (birth weight or birthweight)) IN HTA 18

12 #1 OR #2 OR #3 OR #4 OR #5 OR #6 OR #7 OR #8 OR #9 OR #10 OR #11 2023

13 MeSH DESCRIPTOR Public Health IN HTA 38

14 MeSH DESCRIPTOR Health Promotion IN HTA 70

15 MeSH DESCRIPTOR Health Literacy IN HTA 6

16 MeSH DESCRIPTOR Health Education IN HTA 30

17 MeSH DESCRIPTOR Social Determinants of Health IN HTA 0

18 MeSH DESCRIPTOR Child Health IN HTA 2

19 MeSH DESCRIPTOR Child Development IN HTA 9

20 MeSH DESCRIPTOR Child Guidance IN HTA 1

21 MeSH DESCRIPTOR Child Welfare IN HTA 8

22 MeSH DESCRIPTOR Child Abuse IN HTA 11

23 MeSH DESCRIPTOR Child Nutrition Disorders IN HTA 1

24 MeSH DESCRIPTOR Infant Health IN HTA 0

25 MeSH DESCRIPTOR Infant Welfare IN HTA 5

26 MeSH DESCRIPTOR Family Relations EXPLODE ALL TREES IN HTA 20

27 MeSH DESCRIPTOR Early Intervention, Educational IN HTA 0

28 MeSH DESCRIPTOR Early Medical Intervention IN HTA 1

29 MeSH DESCRIPTOR Physical Fitness EXPLODE ALL TREES IN HTA 9

30 MeSH DESCRIPTOR Diet, Healthy IN HTA 0

31 MeSH DESCRIPTOR Oral Health IN HTA 7

32 ((early or early-years or early-life*) NEAR3 (program* or interven* or scheme* or initiative*)) IN HTA 42

33 ((health* or wellness or welfare or well-being or wellbeing or safety or immuni*) NEAR5 (program* or interven* or scheme* or initiative* or encourag* or promot* or educat* or literacy or campaign* or improve* or improving)) IN HTA 2721

34 ((lifestyle* or diet* or food* or nutrition*) NEAR3 (intervention* or program* or chang* or modif* or improv* or enhanc* or adapt* or target* or alter* or impact*)) IN HTA 104

35 ((behavior* or behaviour* or positive* or success*) NEAR3 (intervention* or program* or chang* or modif* or improv* or enhanc* or adapt* or impact*)) IN HTA 231

36 ((increas* or improv* or encourag* or support* or promot* or influen* or recommend* or motivat* or incentiv* or market* or advert* or subsid* or reward* or persua* or convinc* or instigat* or invest or benefit* or uptak* or start*) NEAR4 ((active or physically-active or health*) NEAR3 (living or life*))) IN HTA 29

37 ((increas* or improv* or encourag* or support* or promot* or influen* or recommend* or motivat* or incentiv* or market* or advert* or subsid* or reward* or persua* or convinc* or instigat* or invest or benefit* or uptak* or start*) NEAR4 (exercise* or exercising or fitness)) IN HTA 51

38 ((decreas* or minimis* or reduc* or discourag* or disincentiv* or dissuade* or deter* or prevent* or avert* or divert) NEAR4 (physical* NEAR2 inactiv*)) IN HTA 0

39 ((decreas* or minimis* or reduc* or discourag* or disincentiv* or dissuade* or deter* or prevent* or avert* or divert) NEAR4 ((inactiv* or unhealthy) NEAR3 (living or life*))) IN HTA 0

40 ((increas* or improv* or encourag* or support* or promot* or influen* or recommend* or motivat* or incentiv* or market* or advert* or subsid* or reward* or persua* or convinc* or instigat* or invest or benefit* or uptak* or start*) NEAR4 ((weight or body mass or BMI) NEAR2 (healthy or manage* or control* or loss* or loos* or decreas* or reduc*))) IN HTA 23

41 ((decreas* or minimis* or reduc* or discourag* or disincentiv* or dissuade* or deter* or prevent* or avert* or divert) NEAR4 (obese or obesity or overweight)) IN HTA 36

42 ((increas* or improv* or encourag* or support* or promot* or influen* or recommend* or motivat* or incentiv* or market* or advert* or subsid* or reward* or persua* or convinc* or instigat* or invest or benefit* or uptak* or start*) NEAR4 ((health* or balanced) NEAR2 (diet* or eating or food or nutrition*))) IN HTA 9

43 ((increas* or improv* or encourag* or support* or promot* or influen* or recommend* or motivat* or incentiv* or market* or advert* or subsid* or reward* or persua* or convinc* or instigat* or invest or benefit* or uptak* or start*) NEAR4 ((salt or sugar or calorie*) NEAR2 (less or lessen or reduc* or restrict*))) IN HTA 0

44 ((decreas* or minimis* or reduc* or discourag* or disincentiv* or dissuade* or deter* or prevent* or avert* or divert) NEAR4 ((fizzy or sugary or sweetened) NEAR2 (drink or beverage*))) IN HTA 0

45 ((breastfeed* or feeding) NEAR3 (advice or advis* or educat* or support*)) IN HTA 5

46 ((decreas* or minimis* or reduc* or discourag* or disincentiv* or dissuade* or deter* or prevent* or avert* or divert) NEAR4 (malnutrition or malnourish* or undernourish* or overnutrition)) IN HTA 1

47 ((increas* or improv* or encourag* or support* or promot* or influen* or recommend* or motivat* or incentiv* or market* or advert* or subsid* or reward* or persua* or convinc* or instigat* or invest or benefit* or uptak* or start*) NEAR4 ((oral* or dental*) NEAR2 (health* or care or hygien*))) IN HTA 8

48 ((decreas* or minimis* or reduc* or discourag* or disincentiv* or dissuade* or deter* or prevent* or avert* or divert) NEAR4 ((oral* or dental* or tooth or teeth) NEAR2 (decay* or disease*))) IN HTA 5

49 ((identif* or detect* or prevent*) NEAR3 ((domestic* or spousal or child* or caregiver* or care-giver* or parent* or maternal* or paternal* or physical* or emotional*) NEAR2 (neglect* or abuse* or abusive or violen* or harm or maltreat* or mistreat*))) IN HTA 3

50 #13 OR #14 OR #15 OR #16 OR #17 OR #18 OR #19 OR #20 OR #21 OR #22 OR #23 OR #24 OR #25 OR #26 OR #27 OR #28 OR #29 OR #30 OR #31 OR #32 OR #33 OR #34 OR #35 OR #36 OR #37 OR #38 OR #39 OR #40 OR #41 OR #42 OR #43 OR #44 OR #45 OR #46 OR #47 OR #48 OR #49 3042

51 #12 AND #50 583

52 * IN HTA FROM 2000 TO 2018 14815

53 #51 AND #52 447

**Key:**

MeSH DESCRIPTOR = indexing term: Medical Subject Heading (MeSH)

EXPLODE ALL TREES = exploded indexing term (MeSH)

* = truncation

NEAR3 = terms within three words of each other (only in the order specified).

**Science Citation Index Expanded**

via Web of Science, Clarivate Analytics https://clarivate.com/

Date range searched: 1900 – 16^th^ August 2021

Date searched: 16^th^ August 2021

Records retrieved: 4369

The SCIE strategy below includes the CADTH economics search filter for Ovid Medline (lines 26-33) and the NICE UK search filter for Ovid Medline (lines 38-52), both of which were adapted for use on this database.

Economic Evaluations/Cost/Economic Models - Ovid Medline. Strings attached: CADTH database search filters [Internet]. Ottawa: CADTH; 2016. [Accessed: 16 August 2021]. Available from: https://www.cadth.ca/resources/finding-evidence/strings-attached-cadths-database-search-filters#health

Ayiku L, Levay P, Hudson T, Craven J, Barrett E, Finnegan A and Adams R. The MEDLINE UK filter: development and validation of a geographic search filter to retrieve research about the UK from OVID MEDLINE. Health Information and Libraries Journal, 2017 34 (3): 200-216. (Publisher: Wiley. © 2017 Crown copyright. Health Information and Libraries Journal © 2017 Health Libraries Group.)

# 55 4,369

#53 NOT #54

Indexes=SCI-EXPANDED Timespan=2000-2021

# 54 2,806,814

TS=(rat or rats or rodent* or mouse or mice or "mus musculus" or "mus domesticus" or murine or murinae or porcine or sheep or ovine or "ovis aries" or lamb or lambs or ewe or ewes or pig or pigs or piglet or piglets or sow or sows or minipig or minipigs or monkey or monkeys or bovine or cattle or heifer or heifers or chicken or chickens or livestock or alpaca* or llama*) Indexes=SCI-EXPANDED Timespan=2000-2021

# 53 4,450

#37 AND #52

Indexes=SCI-EXPANDED Timespan=2000-2021

# 52 3,683,748

#38 OR #39 OR #40 OR #41 OR #42 OR #43 OR #44 OR #45 OR #46 OR #47 OR #48 OR #49 OR #50 OR #51

Indexes=SCI-EXPANDED Timespan=2000-2021

# 51 27,656

AB=(english not ((published or publication* or translat* or written or language* or speak* or literature or citation*) NEAR/5 english))

Indexes=SCI-EXPANDED Timespan=2000-2021

# 50 38,053

OO=(armagh or "armagh's" or belfast or "belfast's" or lisburn or "lisburn's" or londonderry or "londonderry's" or derry or "derry's" or newry or "newry's")

Indexes=SCI-EXPANDED Timespan=2000-2021

# 49 49,445

CI=(armagh or "armagh's" or belfast or "belfast's" or lisburn or "lisburn's" or londonderry or "londonderry's" or derry or "derry's" or newry or "newry's")

Indexes=SCI-EXPANDED Timespan=2000-2021

# 48 227,450

OO=(aberdeen or "aberdeen's" or dundee or "dundee's" or edinburgh or "edinburgh's" or glasgow or "glasgow's" or inverness or (perth not australia*) or ("perth's" not australia*) or stirling or "stirling's")

Indexes=SCI-EXPANDED Timespan=2000-2021

# 47 378,162

CI=(aberdeen or "aberdeen's" or dundee or "dundee's" or edinburgh or "edinburgh's" or glasgow or "glasgow's" or inverness or (perth not australia*) or ("perth's" not australia*) or stirling or "stirling's")

Indexes=SCI-EXPANDED Timespan=2000-2021

# 46 78,169

OO=(bangor or "bangor's" or cardiff or "cardiff's" or newport or "newport's" or "st asaph" or "st asaph's" or "st davids" or swansea or "swansea's")

Indexes=SCI-EXPANDED Timespan=2000-2021

# 45 110,138

CI=(bangor or "bangor's" or cardiff or "cardiff's" or newport or "newport's" or "st asaph" or "st asaph's" or "st davids" or swansea or "swansea's")

Indexes=SCI-EXPANDED Timespan=2000-2021

# 44 1,610,636

OO=(bath or "bath's" or ((birmingham not alabama*) or ("birmingham's" not alabama*) or bradford or "bradford's" or brighton or "brighton's" or bristol or "bristol's" or carlisle* or "carlisle's" or (cambridge not (massachusetts* or boston* or harvard*) ) or ("cambridge's" not (massachusetts* or boston* or harvard*) ) or (canterbury not zealand*) or ("canterbury's" not zealand*) or chelmsford or "chelmsford's" or chester or "chester's" or chichester or "chichester's" or coventry or "coventry's" or derby or "derby's" or (durham not (carolina* or nc) ) or ("durham's" not (carolina* or nc) ) or ely or "ely's" or exeter or "exeter's" or gloucester or "gloucester's" or hereford or "hereford's" or hull or "hull's" or lancaster or "lancaster's" or leeds* or leicester or "leicester's" or (lincoln not nebraska*) or ("lincoln's" not nebraska*) or (liverpool not ("new south wales*" or nsw) ) or ("liverpool's" not ("new south wales*" or nsw) ) or ((london not (ontario* or ont or toronto*) ) or ("london's" not (ontario* or ont or toronto*) ) or manchester or "manchester's" or (newcastle not ("new south wales*" or nsw) ) or ("newcastle's" not ("new south wales*" or nsw) ) or norwich or "norwich's" or nottingham or "nottingham's" or oxford or "oxford's" or peterborough or "peterborough's" or plymouth or "plymouth's" or portsmouth or "portsmouth's" or preston or "preston's" or ripon or "ripon's" or salford or "salford's" or salisbury or "salisbury's" or sheffield or "sheffield's" or southampton or "southampton's" or "st albans" or stoke or "stoke's" or sunderland or "sunderland's" or truro or "truro's" or wakefield or "wakefield's" or wells or westminster or "westminster's" or winchester or "winchester's" or wolverhampton or "wolverhampton's" or (worcester not (massachusetts* or boston* or harvard*) ) or ("worcester's" not (massachusetts* or boston* or harvard*) ) or (york not ("new york*" or ny or ontario* or ont or toronto*) ) or ("york's" not ("new york*" or ny or ontario* or ont or toronto*) ))))

Indexes=SCI-EXPANDED Timespan=2000-2021

# 43 2,625,234

CI=(bath or "bath's" or ((birmingham not alabama*) or ("birmingham's" not alabama*) or bradford or "bradford's" or brighton or "brighton's" or bristol or "bristol's" or carlisle* or "carlisle's" or (cambridge not (massachusetts* or boston* or harvard*) ) or ("cambridge's" not (massachusetts* or boston* or harvard*) ) or (canterbury not zealand*) or ("canterbury's" not zealand*) or chelmsford or "chelmsford's" or chester or "chester's" or chichester or "chichester's" or coventry or "coventry's" or derby or "derby's" or (durham not (carolina* or nc) ) or ("durham's" not (carolina* or nc) ) or ely or "ely's" or exeter or "exeter's" or gloucester or "gloucester's" or hereford or "hereford's" or hull or "hull's" or lancaster or "lancaster's" or leeds* or leicester or "leicester's" or (lincoln not nebraska*) or ("lincoln's" not nebraska*) or (liverpool not ("new south wales*" or nsw) ) or ("liverpool's" not ("new south wales*" or nsw) ) or ((london not (ontario* or ont or toronto*) ) or ("london's" not (ontario* or ont or toronto*) ) or manchester or "manchester's" or (newcastle not ("new south wales*" or nsw) ) or ("newcastle's" not ("new south wales*" or nsw) ) or norwich or "norwich's" or nottingham or "nottingham's" or oxford or "oxford's" or peterborough or "peterborough's" or plymouth or "plymouth's" or portsmouth or "portsmouth's" or preston or "preston's" or ripon or "ripon's" or salford or "salford's" or salisbury or "salisbury's" or sheffield or "sheffield's" or southampton or "southampton's" or "st albans" or stoke or "stoke's" or sunderland or "sunderland's" or truro or "truro's" or wakefield or "wakefield's" or wells or westminster or "westminster's" or winchester or "winchester's" or wolverhampton or "wolverhampton's" or (worcester not (massachusetts* or boston* or harvard*) ) or ("worcester's" not (massachusetts* or boston* or harvard*) ) or (york not ("new york*" or ny or ontario* or ont or toronto*) ) or ("york's" not ("new york*" or ny or ontario* or ont or toronto*) ))))

Indexes=SCI-EXPANDED Timespan=2000-2021

# 42 233,425

SO=(gb or "g.b." or britain* or (british* not "british columbia") or uk or "u.k." or "united kingdom*" or (england* not "new england") or "northern ireland*" or "northern irish*" or scotland* or scottish* or ((wales or "south wales") not "new south wales") or welsh*)

Indexes=SCI-EXPANDED Timespan=2000-2021

# 41 2,587,707

CU=(gb or "g.b." or britain* or (british* not "british columbia") or uk or "u.k." or "united kingdom*" or (england* not "new england") or "northern ireland*" or "northern irish*" or scotland* or scottish* or ((wales or "south wales") not "new south wales") or welsh*)

Indexes=SCI-EXPANDED Timespan=2000-2021

# 40 328,127

TS=(gb or "g.b." or britain* or (british* not "british columbia") or uk or "u.k." or "united kingdom*" or (england* not "new england") or "northern ireland*" or "northern irish*" or scotland* or scottish* or ((wales or "south wales") not "new south wales") or welsh*)

Indexes=SCI-EXPANDED Timespan=2000-2021

# 39 190,502

OO=("national health service*" or nhs*)

Indexes=SCI-EXPANDED Timespan=2000-2021

# 38 29,379

TS=("national health service*" or nhs*)

Indexes=SCI-EXPANDED Timespan=2000-2021

# 37 19,304

#25 AND #36

Indexes=SCI-EXPANDED Timespan=2000-2021

# 36 1,847,560

#26 OR #27 OR #28 OR #29 OR #30 OR #31 OR #32 OR #33 OR #34 OR #35

Indexes=SCI-EXPANDED Timespan=2000-2021

# 35 12,023

TS=(SROI or ROI)

Indexes=SCI-EXPANDED Timespan=2000-2021

# 34 5,514

TS=(return NEAR/3 investment*)

Indexes=SCI-EXPANDED Timespan=2000-2021

# 33 59,385

TS=(decision* NEAR/2 (tree* or analy* or model*) )

Indexes=SCI-EXPANDED Timespan=2000-2021

# 32 195,759

TS=("monte carlo")

Indexes=SCI-EXPANDED Timespan=2000-2021

# 31 83,964

TS=(markov)

Indexes=SCI-EXPANDED Timespan=2000-2021

# 30 7,568

TS=("economic model*")

Indexes=SCI-EXPANDED Timespan=2000-2021

# 29 3,653

TS=(value NEAR/2 (money or monetary) )

Indexes=SCI-EXPANDED Timespan=2000-2021

# 28 295,528

TS=(cost* NEAR/2 (effective* or utilit* or benefit* or minimi* or analy* or outcome or outcomes) )

Indexes=SCI-EXPANDED Timespan=2000-2021

# 27 1,459,439

AB=(economic* or cost or costs or costly or costing or price or prices or pricing or pharmacoeconomic* or pharmaco-economic* or expenditure or expenditures or expense or expenses or financial or finance or finances or financed)

Indexes=SCI-EXPANDED Timespan=2000-2021

# 26 77,051

TS=(budget*)

Indexes=SCI-EXPANDED Timespan=2000-2021

# 25 153,267

#4 AND #24

Indexes=SCI-EXPANDED Timespan=2000-2021

# 24 953,605

#5 OR #6 OR #7 OR #8 OR #9 OR #10 OR #11 OR #12 OR #13 OR #14 OR #15 OR #16 OR #17 OR #18 OR #19 OR #20 OR #21 OR #22 OR #23

Indexes=SCI-EXPANDED Timespan=2000-2021

# 23 1,267

TS=((identif* or detect* or prevent*) NEAR/3 ((domestic* or spousal or child* or caregiver* or care-giver* or parent* or maternal* or paternal* or physical* or emotional*) NEAR/2 (neglect* or abuse* or abusive or violen* or harm or maltreat* or mistreat*) ))

Indexes=SCI-EXPANDED Timespan=2000-2021

# 22 1,220

TS=((decreas* or minimis* or reduc* or discourag* or disincentiv* or dissuade* or deter* or prevent* or avert* or divert) NEAR/4 ((oral* or dental* or tooth or teeth) NEAR/2 (decay* or disease*) ))

Indexes=SCI-EXPANDED Timespan=2000-2021

# 21 6,329

TS=((increas* or improv* or encourag* or support* or promot* or influen* or recommend* or motivat* or incentiv* or market* or advert* or subsid* or reward* or persua* or convinc* or instigat* or invest or benefit* or uptak* or start*) NEAR/4 ((oral* or dental*) NEAR/2 (health* or care or hygien*) ))

Indexes=SCI-EXPANDED Timespan=2000-2021

# 20 3,053

TS=((decreas* or minimis* or reduc* or discourag* or disincentiv* or dissuade* or deter* or prevent* or avert* or divert) NEAR/4 (malnutrition or malnourish* or undernourish* or overnutrition) )

Indexes=SCI-EXPANDED Timespan=2000-2021

# 19 5,911

TS=((breastfeed* or feeding) NEAR/3 (advice or advis* or educat* or support*) )

Indexes=SCI-EXPANDED Timespan=2000-2021

# 18 499

TS=((decreas* or minimis* or reduc* or discourag* or disincentiv* or dissuade* or deter* or prevent* or avert* or divert) NEAR/4 ((fizzy or sugary or sweetened) NEAR/2 (drink or beverage*) ))

Indexes=SCI-EXPANDED Timespan=2000-2021

# 17 3,209

TS=((increas* or improv* or encourag* or support* or promot* or influen* or recommend* or motivat* or incentiv* or market* or advert* or subsid* or reward* or persua* or convinc* or instigat* or invest or benefit* or uptak* or start*) NEAR/4 ((salt or sugar or calorie*) NEAR/2 (less or lessen or reduc* or restrict*) ))

Indexes=SCI-EXPANDED Timespan=2000-2021

# 16 13,072

TS=((increas* or improv* or encourag* or support* or promot* or influen* or recommend* or motivat* or incentiv* or market* or advert* or subsid* or reward* or persua* or convinc* or instigat* or invest or benefit* or uptak* or start*) NEAR/4 ((health* or balanced) NEAR/2 (diet* or eating or food or nutrition*) ))

Indexes=SCI-EXPANDED Timespan=2000-2021

# 15 33,811

TS=((decreas* or minimis* or reduc* or discourag* or disincentiv* or dissuade* or deter* or prevent* or avert* or divert) NEAR/4 (obese or obesity or overweight) )

Indexes=SCI-EXPANDED Timespan=2000-2021

# 14 28,758

TS=((increas* or improv* or encourag* or support* or promot* or influen* or recommend* or motivat* or incentiv* or market* or advert* or subsid* or reward* or persua* or convinc* or instigat* or invest or benefit* or uptak* or start*) NEAR/4 ((weight or "body mass" or BMI) NEAR/2 (healthy or manage* or control* or loss* or loos* or decreas* or reduc*) ))

Indexes=SCI-EXPANDED Timespan=2000-2021

# 13 176

TS=((decreas* or minimis* or reduc* or discourag* or disincentiv* or dissuade* or deter* or prevent* or avert* or divert) NEAR/4 ((inactiv* or unhealthy) NEAR/3 (living or life*) ))

Indexes=SCI-EXPANDED Timespan=2000-2021

# 12 421

TS=((decreas* or minimis* or reduc* or discourag* or disincentiv* or dissuade* or deter* or prevent* or avert* or divert) NEAR/4 (physical* NEAR/2 inactiv*) )

Indexes=SCI-EXPANDED Timespan=2000-2021

# 11 72,277

TS=((increas* or improv* or encourag* or support* or promot* or influen* or recommend* or motivat* or incentiv* or market* or advert* or subsid* or reward* or persua* or convinc* or instigat* or invest or benefit* or uptak* or start*) NEAR/4 (exercise* or exercising or fitness) )

Indexes=SCI-EXPANDED Timespan=2000-2021

# 10 17,365

TS=((increas* or improv* or encourag* or support* or promot* or influen* or recommend* or motivat* or incentiv* or market* or advert* or subsid* or reward* or persua* or convinc* or instigat* or invest or benefit* or uptak* or start*) NEAR/4 ((active or physically-active or health*) NEAR/3 (living or life*) ))

Indexes=SCI-EXPANDED Timespan=2000-2021

# 9 54

TS=("best start in life" or "healthy child programme" or "healthy start programme" or "change for children")

Indexes=SCI-EXPANDED Timespan=2000-2021

# 8 320,436

TS=((behavior* or behaviour* or positive* or success*) NEAR/3 (intervention* or program* or chang* or modif* or improv* or enhanc* or adapt* or impact*) )

Indexes=SCI-EXPANDED Timespan=2000-2021

# 7 178,864

TS=((lifestyle* or diet* or food* or nutrition*) NEAR/3 (intervention* or program* or chang* or modif* or improv* or enhanc* or adapt* or target* or alter* or impact*) )

Indexes=SCI-EXPANDED Timespan=2000-2021

# 6 352,241

TS=((health* or wellness or welfare or well-being or wellbeing or safety or immuni*) NEAR/5 (program* or interven* or scheme* or initiative* or encourag* or promot* or educat* or literacy or campaign* or improve* or improving) )

Indexes=SCI-EXPANDED Timespan=2000-2021

# 5 43,369

TS=((early or early-years or early-life*) NEAR/3 (program* or interven* or scheme* or initiative*) )

Indexes=SCI-EXPANDED Timespan=2000-2021

# 4 1,995,254

#1 OR #2 OR #3

Indexes=SCI-EXPANDED Timespan=2000-2021

# 3 231

TS=("birth to 5" or "birth to five")

Indexes=SCI-EXPANDED Timespan=2000-2021

# 2 3,651

TS=(under NEAR/1 (five* or "5") NEAR/2 (age* or old*) )

Indexes=SCI-EXPANDED Timespan=2000-2021

# 1 1,995,058

TS=(pediatric* or paediatric* or child* or preemie* or baby or babies or infant* or toddler* or "neo nat*" or neo-nat* or neonat* or newborn* or new-born* or "newly born*" or newly-born* or preschool* or pre-school* or schoolchild* or school-child* or schoolboy* or school-boy* or schoolgirl* or school-girl* or school-age* or prekindergarten or pre-kindergarten or kindergarten or boy* or girl* or kid* or LBW or VLBW or ELBW or "low birth weight")

Indexes=SCI-EXPANDED Timespan=2000-2021

**Key:**

TS= terms in either title, abstract, author keywords, and keywords plus fields

TI= search in title field

AB= search in abstract field

CU= search in country/region field

SO= search in publication name field

CI= search in city field

OO= search in organization field

NEAR/3  = terms within three words of each other (any order).

* = truncation

**Appendix 2: Data extraction template**

| **1) General** |
| --- |
| Author |
| Year |
| Target population |
|  |
| **2) Intervention** |
| Intervention being evaluated |
| Comparator (counterfactual) |
| Study design |
| Study location |
| Length of follow-up of study |
|  |
| **3) Economic evaluation (general)** |
| Evaluative framework used (e.g. CEA, CBE, SROI) |
| Perspective |
| Time horizon |
| Discount rate |
|  |
| **3b) Costs** |
| Extent of resource use captured - health and non-health |
| Source of the cost data |
| Did the evaluation capture the opportunity costs? If so, what OC was used? |
|  |
| **3c) Outcomes** |
| Health outcomes captured |
| Any outcomes beyond health? (e.g. educational, child development etc.) if so how were they measured? |
|  |
| **3d) Incoporation of equity considerations** |
| Was there a formal (quantifiable) incorporation of equity considerations? |
| If so, what approach was taken? |
|  |
| **4) Modelling** |
| Was decision analytic modelling used? |
| Structural assumptions |
|  |
| **5) Recommendation** |
| Was the intervention considered to be cost-effective? |
| If cross sectoral outcomes included, how were they combined/traded off in decision making? |
| If equity informative outcomes included, how were equity/efficiency outcomes combined/traded off in decision making? |
|  |
| **6) Characterisation of uncertainty** |
| Was the uncertainty in the structural assumptions explored? |
| Was parameter uncertainty specified? |
| Were distributions used around parametric extrapolations |
| Probabilistic sensitivity analysis results presented? |
| How was uncertainty presented? |
| Did it influence the conclusion? |
|  |
| **Empirical Results** |
| Result of the evaluation (e.g. £/QALY) |
| Disaggregated costs and outcomes: |
| Intervention costs |
| Comparator costs |
| Intervention outcomes |
| Comparator outcomes |

Appendix 3 – Drummond Checklist

| **1** | **Was a well defined question posed in an answerable form?** |
| --- | --- |
| 1.1 | Did the study examine both costs and effects of the service(s) or programme(s) over an appropriate time horizon? |
| 1.2 | Did the study involve a comparison of alternatives? |
| 1.3 | Was a perspective for the analysis stated and was the study placed in any particular decision-making context? |
| 1.4 | Were the patient population and any relevant subgroups adequately defined? |
| **2** | **Was a comprehensive description of the competing alternatives given?** |
| 2.1 | Were any relevant alternatives omitted? |
| 2.2 | Was (should) a 'do nothing' alternative (be) considered? |
| 2.3 | Were relevant alternatives identified for the patient subgroup? |
| **3** | **Was the effectiveness of the programmes or services established?** |
| 3.1 | Was this done thorugh an RCT? If so, did the trial protocol reflect what would happen in regular practice? |
| 3.2 | Were effectiveness data collected and summarized through a systematic overview of clinical studies? If so, were the search strategy and rules for inclusion or exclusion outlined? |
| 3.3 | Were observational data or assumptions used to establish effectiveness? If so, were any potential biases recognized? |
| **4** | **Were all the important and relevant costs and consequences for each alternative identified?** |
| 4.1 | Was the range wide enough for the research question at hand? |
| 4.2 | Did it cover all relevant perspectives? |
| 4.3 | Were capital costs, as well as operating costs, included? |
| **5** | **Were costs and consequences measured accurately in appropriate physical units prior to valuation (e.g. hours of nursing time, number of physician visits, lost work-days, gained life years)?** |
| 5.1 | Were the sources of resource utilisation described and justified? |
| 5.2 | Were any of the identified items omitted from measurement? If so, does this mean that they carried no weight in the subsequent analysis? |
| 5.3 | Were there any special circumstances (e.g. joint use of resources) that made measurement difficult? Were these circumstances handled appropriately? |
| **6** | **Were costs and consequences valued credibly?** |
| 6.1 | Were the sources of all value clearly identified? |
| 6.2 | Were market values employed for changes involving resource gained or depleted? |
| 6.3 | Where market values were absent (e.g. volunteer labour), or market values did not reflect actual values (e.g. clinic space donated at a reduced rate), were adjustments made to approximate market value? |
| 6.4 | Was the valuation of consequences appropriate for the question posed (i.e. has the appropriate type of types of analysis - cost-effectiveness, cost-benefit - been selected)? |
| **7** | **Were costs and consequences adjusted for differential timing?** |
| 7.1 | Were costs and consequences that occur in the future 'discounted' to their present values? |
| 7.2 | Was a justification given for the discount rate used? |
| **8** | **Was an incremental analysis of costs and consequences of alternatives performed?** |
| 8.1 | Were the additional (incremental) costs generated by one alternative over another compared to the additional effects, benefits or utilities generated? |
| **9** | **Was uncertainty in the estimates of costs and consequences adequately characterized?** |
| 9.1 | If patient-level data on costs or consequences were available, were appropriate statistical analyses performed? |
| 9.2 | If a sensitivity analysis was performed, was justification provided for the form(s) of sensitivity analysis employed and the ranges or distributions of values (for key study parameters)? |
| 9.3 | Were the conclusions of the study sensitive to the uncertainty in the results, as quantified by the statistical and/or sensitivity analysis? |
| 9.4 | Was heterogeneity in the patient population recognized, for example by presenting study results for relevant subgroups? |
| **10** | **Did the presentation and discussion of study results include all issues of concern to users?** |
| 10.1 | Were the conclusions of the analysis based on some overall index or ratio of costs to consequences (e.g. cost-effectiveness ratio)? If so, was the index interpreted intelligently or in a mechanistic fashion? |
| 10.2 | Were the results compared with those of others who have investigated the same question? If so, were allowances made for potential differences in study methodology? |
| 10.3 | Did the study discuss the generalizability of the results to other settings and patient/client groups? |
| 10.4 | Did the study allude to, or take account of, other important factors in the choice or decision under consideration (e.g. distribution of costs and consequences, or relevant ethical issues)? |
| 10.5 | Did the study discuss issues of implementation, such as feasibility of adopting the preferred programme given existing financial or other constraints, and whether any freed resources could be redeployed to other worthwhile programmes? |
| 10.6 | Were the implications of uncertainty for decision making,including the need for future research, explored? |

**Appendix 4 – Drummond Checklist Results**

|  | **Drummond Checklist (Y, N, unclear, n/a)** | Achana 2016 | Anokye 2020 | Atkins 2012 | Baguelin 2015 | Bamford 2007 | Barber 2015 | Barlow 2019 | Barnardo's 2012a | Barnardo's 2012b | Beck 2021 | Bessey 2019 | Bessey 2018 | Boyd 2016 | Brisson 2003 |
| --- | --- | --- | --- | --- | --- | --- | --- | --- | --- | --- | --- | --- | --- | --- | --- |
| **1** | Was a well defined question posed in an answerable form? | Y | Y | Y | Y | Y | Y | Y | Y | Y | Y | Y | Y | Y | Y |
| **2** | Was a comprehensive description of the competing alternatives given? | Y | Y | Y | Y | Y | Y | Y | N | N | Y | Y | Y | Y | Y |
| **3** | Was the effectiveness of the programmes or services established? | Y | Y | Y | Y | Y | Y | Y | Y | Y | Y | Y | Y | Y | Y |
| **4** | Were all the important and relevant costs and consequences for each alternative identified? | Y | Y | Y | Y | Y | Y | Y | Y | Y | Y | Y | Y | Y | Y |
| **5** | Were costs and consequences measured accurately in appropriate physical units prior to valuation (e.g. hours of nursing time, number of physician visits, lost work-days, gained life years)? | Y | Y | Y | Y | Y | Y | Y | Y | Y | Y | Y | Y | Y | Y |
| **6** | Were costs and consequences valued credibly? | Y | Y | Y | Y | Y | Y | Y | Y | Y | Y | Y | Y | N | Y |
| **7** | Were costs and consequences adjusted for differential timing? | Y | n/a | Y | Y | Y | n/a | n/a | N | N | Y | Y | Y | N | Y |
| **8** | Was an incremental analysis of costs and consequences of alternatives performed? | Y | Y | Y | Y | Y | Y | Y | N | N | Y | Y | Y | N | Y |
| **9** | Was uncertainty in the estimates of costs and consequences adequately characterized? | Y | Y | Y | Y | Y | Y | Y | Y | Y | Y | Y | Y | N | Y |
| **10** | Did the presentation and discussion of study results include all issues of concern to users? | Y | N | Y | Y | N | N | N | N | N | N | Y | Y | N | N |

|  | **Drummond Checklist (Y, N, unclear, n/a)** | Carlton 2008 | Chance 2013 | Christensen 2013 | Christensen 2014 | Craig 2011 | Davenport 2003 | Davies 2003 | Davies 2000 | Edmunds 2002 | Edwards 2007 | Ewer 2012 | Fayter 2007 | Fortnum 2016 | Gardner 2017 |
| --- | --- | --- | --- | --- | --- | --- | --- | --- | --- | --- | --- | --- | --- | --- | --- |
| **1** | Was a well defined question posed in an answerable form? | Y | N | Y | Y | Y | Y | Y | Y | Y | Y | Y | Y | Y | Y |
| **2** | Was a comprehensive description of the competing alternatives given? | Y | N | Y | Y | Y | N | Y | Y | Y | Y | Y | Y | Y | Y |
| **3** | Was the effectiveness of the programmes or services established? | Y | Y | Y | Y | Y | Y | Y | Y | Y | Y | Y | Y | Y | Y |
| **4** | Were all the important and relevant costs and consequences for each alternative identified? | Y | Unclear | Y | Y | Y | Y | Y | Y | Y | Y | Y | Y | Y | Y |
| **5** | Were costs and consequences measured accurately in appropriate physical units prior to valuation (e.g. hours of nursing time, number of physician visits, lost work-days, gained life years)? | Y | Y | Y | Y | Y | Y | Y | Y | Y | Y | Y | Y | Y | Y |
| **6** | Were costs and consequences valued credibly? | Y | Y | Y | Y | Y | N | N | N | Y | Y | Y | Y | Y | Y |
| **7** | Were costs and consequences adjusted for differential timing? | Y | Y | Y | Y | Y | N | N | N | Y | n/a | N | Y | Y | N |
| **8** | Was an incremental analysis of costs and consequences of alternatives performed? | Y | N | Y | Y | Y | Y | Y | Y | Y | Y | Y | Y | Y | Y |
| **9** | Was uncertainty in the estimates of costs and consequences adequately characterized? | Y | Y | Y | Y | Y | Y | N | Y | Y | Y | Y | Y | Y | Y |
| **10** | Did the presentation and discussion of study results include all issues of concern to users? | Y | N | Y | Y | N | N | N | Y | N | Y | N | Y | N | Y |

|  | **Drummond Checklist (Y, N, unclear, n/a)** | Grill 2006 | Hoddinott 2012 | Hodgson 2020 | Hollingworth 2012 | Jacklin 2007 | Jit 2007 | Jit 2009 | Jit 2010 (previous model) | Kay 2018 | Kendrick 2017 i | Kendrick 2017 ii | Knerer 2012 | Knowles 2005 | Kowash 2006 |
| --- | --- | --- | --- | --- | --- | --- | --- | --- | --- | --- | --- | --- | --- | --- | --- |
| **1** | Was a well defined question posed in an answerable form? | Y | Y | Y | Y | N | Y | Y | Y | Y | Y | Y | Y | Y | N |
| **2** | Was a comprehensive description of the competing alternatives given? | Y | Y | Y | Y | N | Y | Y | Y | Y | Y | Y | Y | Y | Y |
| **3** | Was the effectiveness of the programmes or services established? | Y | Y | Y | Y | Unclear | Y | Y | Y | Y | Y | Y | Y | Y | Y |
| **4** | Were all the important and relevant costs and consequences for each alternative identified? | Unclear | unclear | Y | Y | unclear | Y | Y | Y | Y | Y | Y | unclear | unclear | unclear |
| **5** | Were costs and consequences measured accurately in appropriate physical units prior to valuation (e.g. hours of nursing time, number of physician visits, lost work-days, gained life years)? | Y | Y | Y | Y | Y | Y | Y | Y | Y | Y | Y | Y | Y | Y |
| **6** | Were costs and consequences valued credibly? | Y | N | Y | Y | Y | Y | Y | Y | Y | Y | Y | Y | N | N |
| **7** | Were costs and consequences adjusted for differential timing? | Y | n/a | Y | Y | unclear | Y | Y | Y | Y | Y | n/a | Y | unclear | n/a |
| **8** | Was an incremental analysis of costs and consequences of alternatives performed? | Y | Y | Y | Y | Y | Y | Y | Y | Y | Y | Y | Y | Y | Y |
| **9** | Was uncertainty in the estimates of costs and consequences adequately characterized? | Y | N | Y | Y | Y | Y | Y | Y | Y | Y | Y | Y | Y | N |
| **10** | Did the presentation and discussion of study results include all issues of concern to users? | N | N | Y | N | Y | N | N | N | N | Y | N | Y | Y | N |

|  | **Drummond Checklist (Y, N, unclear, n/a)** | Martin 2009 | McAuley 2004 | McIntosh 2003 | Melegaro 2004 | Morell 2000a (& Morrell 2000b) | Mujica 2006 | O'Neill 2017 | Pandor 2004 (& Pandor 2006) | Phillips 2011 | Pokhrel 2015 | Pitman 2013 | Renwick 2018 | Roberts 2012 | Saramago 2014 |
| --- | --- | --- | --- | --- | --- | --- | --- | --- | --- | --- | --- | --- | --- | --- | --- |
| **1** | Was a well defined question posed in an answerable form? | Y | Y | Y | Y | Y | Y | Y | Y | Y | Y | Y | Y | Y | Y |
| **2** | Was a comprehensive description of the competing alternatives given? | Y | Y | Y | Y | Y | Y | Y | Y | Y | Y | Y | Y | Y | Y |
| **3** | Was the effectiveness of the programmes or services established? | Y | Y | Y | Y | Y | Y | Y | Y | Y | Y | Y | Y | Y | Y |
| **4** | Were all the important and relevant costs and consequences for each alternative identified? | Y | Y | Y | Y | Y | Y | Y | Y | Y | Y | Y | Y | Y | Y |
| **5** | Were costs and consequences measured accurately in appropriate physical units prior to valuation (e.g. hours of nursing time, number of physician visits, lost work-days, gained life years)? | Y | Y | Y | Y | Y | Y | Y | Y | Y | Y | Y | Y | Y | Y |
| **6** | Were costs and consequences valued credibly? | Y | Y | Unclear | Y | N | Y | Y | Y | Y | Y | Y | N | Y | Y |
| **7** | Were costs and consequences adjusted for differential timing? | Y | n/a | N | Y | N | N | N | N | n/a | Y | Y | n/a | n/a | Y |
| **8** | Was an incremental analysis of costs and consequences of alternatives performed? | Y | N | Y | Y | Y | Y | Y | Y | Y | Y | Y | Y | Y | Y |
| **9** | Was uncertainty in the estimates of costs and consequences adequately characterized? | Y | N | Y | Y | Y | Y | Y | Y | N | Y | Y | Y | Y | Y |
| **10** | Did the presentation and discussion of study results include all issues of concern to users? | Y | N | N | N | N | Y | Y | Y | Y | N | Y | Y | N | Y |

|  | **Drummond Checklist (Y, N, unclear, n/a)** | **Simkiss 2013** | **Simpson 2005** | **Thomas 2018** | **Tickle 2016** | **Trotter 2002** | **Trotter 2006a** | **Trotter 2006b** | **Tudor Edwards 2016** | **Uus 2006** |
| --- | --- | --- | --- | --- | --- | --- | --- | --- | --- | --- |
| **1** | Was a well defined question posed in an answerable form? | Y | Y | N | Y | N | Y | Y | Y | N |
| **2** | Was a comprehensive description of the competing alternatives given? | Y | Y | Y | Y | Y | Y | Y | Y | Y |
| **3** | Was the effectiveness of the programmes or services established? | Y | Y | Y | Y | Y | Y | Y | Y | unclear |
| **4** | Were all the important and relevant costs and consequences for each alternative identified? | Y | Y | unclear | Y | Y | Y | Y | Y | Y |
| **5** | Were costs and consequences measured accurately in appropriate physical units prior to valuation (e.g. hours of nursing time, number of physician visits, lost work-days, gained life years)? | Y | Y | Y | Y | Y | Y | Y | Y | Y |
| **6** | Were costs and consequences valued credibly? | Y | N | Y | Y | N | Y | N | N | N |
| **7** | Were costs and consequences adjusted for differential timing? | N | Y | N | N | Y | Y | Y | n/a | N |
| **8** | Was an incremental analysis of costs and consequences of alternatives performed? | Y | Y | unclear | Y | Y | Y | Y | Y | Y |
| **9** | Was uncertainty in the estimates of costs and consequences adequately characterized? | Y | Y | Y | Y | Y | Y | Y | Y | N |
| **10** | Did the presentation and discussion of study results include all issues of concern to users? | N | N | N | Y | N | N | N | Y | N |

**Appendix 5 – Results of the economic evaluations**

| **Author, Year** | **Intervention category** | **Universal or targeted** | **Intervention** | **Population** | **Evidence** | **Evaluative framework** | **Perspective** | **Time Horizon** | **Discount rate** | **Extent of costs captured** |
| --- | --- | --- | --- | --- | --- | --- | --- | --- | --- | --- |
| Achana 2016 | Injury prevention | Targeted | Six intervention combinations of education, equipment, home inspection and fitting | Under 4 years | NMA | CUA & CEA | NHS & PSS | 100 years | 3.5% | NHS & PSS |
| Anokye 2020 | Breast feeding | Universal | Nourishing Start for Health (NOSH) | Newborn | RCT | CEA | NHS | 1 year | No discounting | NHS |
| Atkins 2012 | Health protection | Universal | RotaTeq | Under 6 months | RCT | CUA | NHS | 50 years | 3.5% | NHS |
| Baguelin 2015 | Health protection | Universal | LAIV (vaccine) | 2-4 years | SLR | CUA | NHS | 10 years | 3.5% | NHS |
| Bamford 2007 | Hearing/vision screening | Universal and targeted | Alternative SES programmes | 4-5 years | Survey; SLR | CUA | NHS, education services, patients and family | 11 years | 3.5% | Healthcare, social care, education |
| Barber 2015 | Health promotion | Universal | Preschoolers in the Playground (PiP) | 1-4 years | RCT | CUA | NHS | 1 year | No discounting | NHS & PSS |
| Barlow 2019 | Reducing risk of abuse/maltreatment | Targeted | Parents under Pressure (PuP) | Under 2 years | RCT | CUA | NHS & PSS (scenario analysis of societal perspective) | 1 year | No discounting | Health & PSS, legal services and costs borne directly by parents |
| Barnardo's 2012a | Parenting support | Universal and targeted | Barnardo’s Children’s Centre service: Stay and Play | Under 2 years | Qualitative data | SROI | Societal | 5 years | No discounting | Council costs and parent/carer contributions |
| Barnardo's 2012b | Parenting support | Targeted | Barnardo's Children's Centre service: Family Support Worker | Under 5 years | Qualitative data | SROI | Societal | 5 years | No discounting | Council costs, Barnardos and School (venue) costs |
| Beck 2021 | Health protection | Universal | 4CMenB vaccination | Under 1 year | Case-control study; SLR | CUA | NHS + (scenario analysis of societal perspective) | 100 years | 3.5% | NHS, special educational needs costs, productivity losses |
| Bessey 2019 | Newborn screening | Universal | Severe combined immunodeficiency (SCID) screening | Newborn | SLR | CUA | NHS & PSS | 5 years | 3.5% | NHS |
| Bessey 2018 | Newborn screening | Universal | X-ALD screening | Newborn | SLR | CUA | NHS & PSS | Lifetime | 3.5% | NHS & PSS, special education costs |
| Boyd 2016 | Reducing risk of abuse/maltreatment | Targeted | New Orleans-Glasgow model | Under 5 years | Pre-post study; literature; expert opinion | CCA | Societal | 5 years | 3.5% (costs) | NHS, social services, legal system and birth parents productivity losses |
| Brisson 2003 | Health protection | Universal | VZV vaccination (infant strategy) | 12 - 15 months | Epidemiological model | CUA | NHS & societal | 80 years | 3% | Direct medical costs. The societal perspective includes all medical and productivity loss costs as well as household expenditures |
| Burke 2012 | Newborn screening | Universal | i) Universal newborn hearing screening & ii) One-stage universal screening | Newborn | Literature | CEA | NHS + (scenario analysis of societal perspective) | Unclear | No discounting | Health costs, travel time and lost productivity due to symptom-related work absence |
| Carlton 2008 | Hearing/vision screening | Universal | Amblyopia (and stabismus) screening | 3-5 years | SLR | CUA | NHS and "other government departments" | 100 years | 3.5% | NHS |
| Chance 2013 | Parenting support & health promotion | Targeted | Cambridgeshire’s Funded Two-year-old Childcare | 2 years | Questionnaire | SROI | Societal | 5 years | 3.5% (costs) | Local authority costs |
| Christensen 2013 | Health protection | Universal | New 'MenB’ vaccine | 2 months to 4 years | SLR | CUA | NHS & PSS | 100 years | 3.5% for the first 30 years, 3.0% in years 31–75 and 2.5% in years 76–99 | NHS |
| Christensen 2014 | Health protection | Universal | Bexsero | 2 months to 1 year | SLR | CUA | NHS & PSS | 100 years | 3.5% | NHS & PSS, litigation costs falling on the NHS |
| Craig 2011 | Short stature screening | Universal | Grote strategy for short stature screening | Under 3 years | SLR | CUA | NHS & PSS | 12 years | 3.5% | NHS |
| Davenport 2003 | Oral health | Universal | 3-, 6-, 12-, 18-, 24- and 36-month dental check recall policies | 3 months | SLR | CEA | NHS (not explicit) | 6 years | 6% (costs) | NHS |
| Davies 2000 | Newborn screening | Universal and targeted | Neonatal screening nurse follow-up | Newborn | SLR | CEA | NHS (not explicit) | Unclear | 6% (costs) | NHS |
| Davies 2003 | Oral health | Targeted | The provision of free toothpaste and toothbrushes to 3-month | 1 year | RCT | CEA | NHS | 4 years | 5% (costs) | Intervention |
| Edmunds 2002 | Health protection | Universal | Acellular pertussis booster | 4 years | Literature; HES data | CEA | NHS + (scenario analysis of societal perspective) | lifetime | 3% | NHS and prodctivity losses |
| Edwards 2007 | Parenting support | Targeted | The Webster-Stratton Incredible Years basic parenting programme | 3-4 years | RCT | CEA | Societal | 1 year | No discounting | NHS & PSS and special educational services |
| Ewer 2012 | Newborn screening | Universal | Pulse oximetry screening | Newborn | Diagnostic accuracy study | CEA | NHS | 1 year | 3.5% (costs) | NHS |
| Fayter 2007 | Short stature screening | Universal | Short stature screening | 5 years | SLR | CUA | NHS | Lifetime | 3.5% | NHS |
| Fortnum 2016 | Hearing/vision screening | Universal | Hearing screening | 4-5 years | Case-control study; SLR | CUA | NHS & the family | 4 years | 3.5% | NHS and transportation costs for family |
| Gardner 2017 | Parenting support | Targeted | IY Basic parenting programme | 5 years | MA | CEA & ROI | Public sector | 25 years | 3.5% (costs) | NHS, social services departments, Department for Education, voluntary sector, criminal justice system, health impacts of crime and benefits payments |
| Griebsch 2007 | Newborn screening | Universal | Congenital heart defect screening | Newborn | SLR + observational study | CEA | NHS | 1 year | No discounting | NHS |
| Grill 2006 | Hearing/vision screening | Universal | Hearing screening | Newborn | SLR | CEA | NHS | 10 years | 6% costs, 1.5% outcome | NHS |
| Hoddinott 2012 | Breast feeding | Targeted | FEeding Support Team (FEST) | Newborn | RCT | CEA | NHS (not explicit) | 6-8 weeks | No discounting | NHS |
| Hodgson 2020 | Health protection | Universal and targeted | RSV vaccination | Under 5 years | Literature search | CUA | NHS & PSS | 10 years | 3.5% | NHS & PSS |
| Hollingworth 2012 | Health promotion | Targeted | Obesity/overweight interventions | 4-5 years | SLR | CEA | NHS | Lifetime | 3.5% | NHS |
| Jacklin 2007, NICE 2008 | Breast feeding | Targeted | Breast feeding peer support | Newborn | Pre-post study | CUA | NHS (not explicit) | Unclear | 3.5% (only QALYs stated) | NHS and costs of running the sevice |
| Jit 2007 | Health protection | Universal | Rotavirus vaccination | 2-4 months | RCT | CUA & CEA | NHS | Unclear | 3.5% (both) for the first 30 years, 3.0% thereafter | NHS and lost productivity for the care giver |
| Jit 2009 | Health protection | Universal | Rotavirus vaccination | 2-4 months | Literature | CUA | NHS + (scenario analysis of societal perspective) | 5 years | 3% | NHS. Societal costs included NHS, lost productivity for carers and out-of-pocket expenses |
| Jit 2010 (Update of 2009 paper with new efficacy evidence) | Health protection | Universal | Rotavirus vaccination | 2-4 months | RCT | CUA | NHS + (scenario analysis of societal perspective) | 5 years | 3% | NHS. Societal costs included NHS, lost productivity for carers and out-of-pocket expenses |
| Kay 2018 | Oral health | Targeted | Supervised tooth brushing | 5 years | RCT | CUA | States 'public sector' but appears to be NHS | 3 years | 1.5% | NHS |
| Kendrick 2017 i | Injury prevention | Targeted | a) Functional smoke alarm  b) Safe hot tap water temperature  c) Promoting safety gate possession and use  d) Promoting the safe storage of medicines  e) Promoting the safe storage of household and other products | Under 5 years | NMA | CUA | Public sector | 100 years | 3.5% | NHS & PSS and other public sector costs |
| Kendrick 2017 ii | Injury prevention | Targeted | IPB with or without facilitation | Under 3 years | RCT | CEA | Societal | 1 year | No discounting | Children’s centre; fire and rescue service; other agencies including local councils; family costs |
| Knerer 2012 | Health protection | Universal | Pneummococcal vaccination | Under 2 years | Literature | CUA | NHS (not explicit) | 94 years | 3.5% | NHS |
| Knowles 2005 | Newborn screening | Universal | Congenital heart defect screening | Newborn | SLR | CEA | NHS | 1 year | 6% | NHS |
| Kowash 2006 | Oral health | Targeted | Out-reach education programme | Under 1 year | RCT | CBA & CEA | NHS | 3 years | No discounting | NHS |
| Lorgelly 2007 | Health protection | Universal | Rotavirus vaccination programme | Newborn | Literature | CEA | NHS & societal | 5 years | 3.5% | NHS. Societal costs included NHS, lost productivity and OTC medicines |
| Martin 2009 | Health protection |  |  |  |  |  |  |  |  |  |
| McAuley 2004 | Parenting support | Targeted | Home Start support | Under 5 years | Interview (naive comparison) | CEA & CCA | Children and their families | 1 year | No discounting | Education and child care service use, hospital inpatient service use, community health service use, mental health service use, |
| McIntosh 2003 | Health protection | Universal | Pneumococcal vaccination | Under 6 months | RCT | CEA | NHS (includes scenario with lost labour costs to families of children) | 10 years | 6% (costs) | NHS. Included a scenario which incorporated parent's lost productivity |
| Melegaro 2004 | Health protection | Universal | Pneumococcal vaccination | 2 months to 2 years | RCT | CEA | NHS | Lifetime | 3.5% costs, 1.5% benefits | NHS |
| Morell 2000a & Morell 2000b | Parenting support | Universal | Postnatal support from a community midwifery support worker (SW) | Newborn | RCT | CCA (some ambiguity as one SLR referred to it as a cost-analysis but they do report costs and outcomes separately. Could also be a cost-minimisation assuming both have same effect) | NHS | 6 months | 5% costs | NHS |
| Mujica 2006 | Parenting support & health promotion | Targeted | Means-tested access to full-time or part-time day care at the Hackney Early Years Centre | 6 months to 3.5 years | RCT | CEA | Societal | 18 months | 6% costs after 12 months | Early years education and care, NHS, productivity gains and other contributions relating to mothers and their partners. Out-of-pocket costs to parents for travel to health care and child education services and medications |
| O'Neill 2017 | Oral health | Universal | Caries prevention | 2-3 years | RCT | CEA | Public payer | 3 years | No discounting | NHS and lost productivity |
| Pandor 2004 + Pandoor 2006 | Newborn screening | Universal | Inborn errors of metabolism screening | Newborn | SLR | CEA | Health and other public sector providers within the UK' | 80 years | 6% (costs) | NHS & PSS, education sector costs |
| Phillips 2011 | Injury prevention | Targeted | Scald prevention | Under 5 years | RCT | CEA & CBA (but not stated) | Public sector | 1 year | No discounting (the discussion did include a back of the envelope calculation which used 3.5% rate for outcomes) | Public sector costs |
| Pitman 2013 | Health protection | Universal | Influenza vaccination | 2-4 years | Observational study | CUA | NHS | 200 years | 3.5% | NHS |
| Pokhrel 2015 | Breast feeding | Targeted | Breast feeding support | Newborn | SLR; observational study | Not explicit but costs and QALYs are presented albeit doesn't appear to be incremental. Could call it a CCA | NHS | For three acute conditions (GI, LRTI and AOM), analysis was limited to the first year of life; maternal BC took a lifetime horizon; NEC focussed on the stay in a neonatal unit | 3.5% | NHS |
| Renwick 2018 | Health promotion | Targeted | Smoking home intervention | Under 5 years | RCT | CEA | NHS & PSS | 12 weeks | No discounting | NHS |
| Roberts 2012 | Newborn screening | Universal | Congenital heart defect screening | Newborn | RCT | CEA | NHS | 1 year | No discounting | NHS |
| Saramago 2014 | Injury prevention | Universal | Fire injury prevention interventions | Under 5 years | MA | CUA | Public sector, including the NHS and PSS | 100 years | 3.50% | NHS & PSS. Scenario which includes law enforcement and fire and rescue costs |
| Siddiqui 2011 | Health protection | Universal and targeted | HBV programme | Under 6 months | Literature | CUA | NHS | 99 years | 3.50% | NHS |
| Simkiss 2013 | Parenting support | Targeted | The Family Links Nurturing Programme | 2-4 years | RCT | CUA | NHS & PSS | 10 years | No discounting | NHS & PSS |
| Simpson 2005 | Newborn screening | Universal | Cystic Fibrosis screening | Newborn | Literature | CUA | NHS | Lifetime | 6% costs; 2% outcomes | NHS |
| Thomas 2018 | Health protection | Targeted | RSV vaccination | Under 2 years | Observational study | CBA | Societal | Lifetime | 3.5% (costs) | NHS and lost productivity |
| Tickle 2016 | Oral health | Universal | NIC-PIP caries prevention | 2-3 years | RCT | CEA | NHS | 3 years | No discounting | Dental services |
| Trotter 2002 | Health protection | Universal | Meningitis C vaccination | Under 4 years | Literature + observational study | CEA | NHS | Lifetime | 3% | NHS |
| Trotter 2006a | Health protection | Universal | Meningococcal vaccination | Under 1 year | Literature + observational study | CUA & CEA | NHS | 100 years | 3% | NHS |
| Trotter 2006b | Health protection | Universal | Meningococcal vaccination | Under 2 years | Literature + observational study | CEA | NHS | 75 years | 3.50% | NHS |
| Tudor Edwards 2016 | Parenting support | Targeted | IY BASIC parenting programme | 3-4 years | RCT | CEA | Public sector multi-agency | 6 months | No discounting | NHS & PSS and special education services |
| Uus 2006 | Newborn screening | Universal | Newborn Hearing Screening Programme (NHSP) | Newborn | Literature | CEA | Societal | 10 years | No discounting | NHS and costs to the family |
|  |  |  |  |  |  |  |  |  |  |  |

| **Author, Year** | **Outcomes captured** | **Formal incorporation of equity** | **General equity consideration** | **Modelling approach** | **Recommended?** | **Structural uncertainty** | **PSA** | **Reporting of uncertainty in the results** | **Result of the economic evaluation** |
| --- | --- | --- | --- | --- | --- | --- | --- | --- | --- |
| Achana 2016 | QALYs & Numbers of poison cases avoided | Yes. Sensitivity analysis of increasing the rate of unintentional poisoning to the rate observed in the 4^th^ and 5^th^ most deprived quintiles. | Model for under 5-year-olds from socio-economic disadvantaged groups whom the evidence suggest are at increased risk of unintentional injury compared to those from a well-off family background. | Decision tree + markov model | CUA: No interventions were considered cost-effective.  CEA:  No interventions were considered cost-effective. | No | Yes | CE-plane (PSA); CEAC; DSA. | CEA: Compared with usual care, the intervention with the lowest ICER was education at £2888 per poison avoided.  CUA: Compared to usual care, the ICER was lowest for education at £41,330 per QALY gained. |
| Anokye 2020 | Proportion baby breast fed at 6 weeks | No | Intervention focussed in area with low breastfeeding prevalence. | n/a | Not stated. Threshold analysis. | No (trial based evaluation) | Yes | CEAC; DSA | Compared to usual care, the ICER was £974 per additional baby breast fed baby |
| Atkins 2012 | QALYs | No | No | Dynamic transmission model | Yes | No | Yes | CE-plane (PSA); CEAC; DSA | Compared to no vaccination, the ICER using the dynamic model was £27,133 per QALY gained; using the static model was £34,728 per QALY gained.  Other scenarios presented. |
| Baguelin 2015 | QALYs | No | No | Dynamic transmission model | Yes | No | Yes | CE-plane (PSA); DSA | Compared to no vaccination, the ICER is £2,613 per QALY gained |
| Bamford 2007 | QALYs | No | No | Decision tree | Yes | No | Yes | CEAC | Compared with no SES, the NMB is £4867 |
| Barber 2015 | QALYs | No | Intervention implemented in Bradford due to area’s ethnic diversity and social deprivation | n/a | Borderline | No (trial based evaluation) | Yes | CEAC | Compared to usual practice, the ICER is £19,588 per QALY gained |
| Barlow 2019 | QALYs | No | Intervention for substance misusing parents | n/a | Borderline | No (trial based evaluation) | Yes | Probability of being cost effective; DSA | Compared to treatment as usual, the ICER is £34,095 per QALY gained (NHS & PSS perspective); £56,269 per QALY gained (societal perspective) |
| Barnardo's 2012a | Monetary outcomes | No | Stay and Play is considered to be a useful gateway for in need families to access more targeted services. | n/a | Yes - positive SROI | No | No | DSA | Approximately £2 for every £1 invested |
| Barnardo's 2012b | Monetary outcomes | No | The FSW service makes provision for families who are just above the threshold at which social services would intervene. | n/a | Yes - positive SROI | No | No | DSA | £4.50 for every £1 invested |
| Beck 2021 | QALYs | No | No | Dynamic transmission model | Yes | No | Yes | CEAC; DSA | Compared to no vaccination, the ICER is £18 645 per QALY gained |
| Bessey 2019 | QALYs | No | No | Decision tree | Not stated | No | Yes | CE-plane (PSA); DSA. Conducted VOI analysis in the form of EVPI and EVPPI. | Compared to no screening, the ICER is £18,222 per QALY gained |
| Bessey 2018 | QALYs | No | No | Decision tree | Yes | No | Yes | CE-plane (PSA); DSA | Compared to no screening, screening dominates (positive QALYs, negative costs) |
| Boyd 2016 | Probability of one and two episodes in care | No | No | n/a | Not stated | No | No | n/a | Compared to the existing Glasgow model, the New Orleans model has a reduced probability of two episodes in care (incremental reduction of 0.41) and reduced mean cost per child in the model (incremental difference of £6,820). Other outcomes differ in magnitude and direction. |
| Brisson 2003 | QALYs | No | No | Dynamic transmission model | No | No | Yes | CEAC; DSA | Compared to no vaccination, the VZV infant vaccination strategy is dominated as it results in a significant QALY loss. |
| Burke 2012 | Cases detected | No | No | Decision tree | Unclear for NHS perspective, cost saving for societal perspective | No | No | n/a | Compared to selective screening, the ICER is £36,181 per case detected. |
| Carlton 2008 | QALYs | No | No | Markov model | Unlikely to be cost-effective | No | Yes | CEAF (frontier); DSA. Conducted VOI analysis in the form of EVPI. | Compared to no screening, screening at 3 years without autorefraction was the most cost-effective with an ICER of £527,375 per QALY gained. |
| Chance 2013 | Monetary outcomes | No | This was targeted at disadvantaged families | n/a | Positive SROI | No | No | DSA | £8.40 for every £1 invested. |
| Christensen 2013 | QALYs | No | No | Cohort model & dynamic transmission model | No | No | Yes | CEAC | Compared to no vaccination, the ICERs for the various infant strategies ranged from £162,800 to £290,000 per QALY gained (cohort model); and between £91,800 to £97,600 per QALY gained (dynamic model) |
| Christensen 2014 | QALYs | No | No | Dynamic transmission model | No recommendation. Results were presented as the price the vaccine would have to be to be deemed cost-effective at a threshold of £20,000 | No | No | Not presented | Compared to no vaccination, the ICERs for the various infant strategies ranged from £163,100 to £221,000 per QALY gained. |
| Craig 2011 | QALYs | No | No | Decision tree | Yes | No | Yes | CE-plane (PSA); CEAC; DSA | Compared to the UK strategy, the ICER is £1144 per QALY gained |
| Davenport 2003 | Number of teeth free from decay, fillings or extraction | Yes. The cost-effectiveness was evaluated across two groups: those using manual toothbrushing and those using non-manual toothbrushing. These were used as proxies to categorise participants into socioeconomic status. | Key risk factor was used for the present study: socioeconomic background (manual versus nonmanual) | Markov model | Not stated | No | No | n/a | No ICERs reported. |
| Davies 2000 | SCD cases identified | No | Access issues relating to haemoglobinopathy screening, particularly as they relate to race. | Unclear | For areas where there are 16 sickle cell traits and 0.5 sickle cell disease cases per 1000 births, the data suggest that universal screening is cost-effective | No | No | n/a | Compared to targeted screening, range of ICERs reported for various disease incidence rates. For example, prevalence of 0.1 or 0.3 per 1000 births, results in ICERs in the range £25,000– £100,000 per case identified |
| Davies 2003 | Decayed, missing and filled teeth reduction by one unit; child kept free of caries experience; child kept free of extraction experience | No | The intervention was for children living in deprived, non-fluoridated areas of North-West England. | n/a | Not stated | No | No | n/a | Compared to doing nothing, ICERs are £80.83 per tooth saved from carious attack; £424.38 per child kept free of caries experience; £679.01 per extraction avoided |
| Edmunds 2002 | Life-years gained; general practitioner consultation; and hospitalisation averted | No | No | Dynamic transmission model | Not possible to draw any strong conclusions regarding the cost-effectiveness of acellular booster doses from the perspective of the health care provider | No | Yes | No probabilistic results presented graphically; DSA | Compared to no vaccination the range of ICERs reported for various booster doses ranges from £8,463 to ££49,511 per life year gained from the health perspective; £2,489 to £36,941 per life year gained from a societal perspective. |
| Edwards 2007 | ECBI-I | No | The intervention was given to families who were mostly socially and economically disadvantaged compared with the mean values for the UK. | n/a | Likely to be cost effective for a ceiling ratio of £100 per point increase in intensity score. Threshold analysis. | No (trial based evaluation) | Yes | CE-plane (PSA); CEAC; DSA | Compared to a six-month waiting list, the ICER is £71 per 1 point change in the ECBI-I score |
| Ewer 2012 | Detection of CHD | No | No | Decision tree | If society’s WTP would be £50,000 then the probability that pulse oximetry as an adjunct to clinical examination’ is cost-effective is >90%. Threshold analysis. | No | Yes | CE-plane (PSA); CEAC | Compared to clinical examination alone, the ICER is £24,900 per timely diagnosis |
| Fayter 2007 | QALYs | No | No | Decision tree | Yes | No | Yes | CE-plane (PSA); CEAC. Planned to do VOI analysis but it was not undertaken. | Compared to no monitoring, the ICER is £9500 per QALY gained |
| Fortnum 2016 | QALYs | No | No | Decision tree | Unlikely to be cost-effective | No | No | n/a | Compared to no screening, the SES programme is dominated |
| Gardner 2017 | ECBI-I | No | The study was concerned with distributional impacts across groups. However, there were no differential effects of IY on disruptive behaviour in families with different levels of social/socioeconomic disadvantage or differential effects for ethnic minority families, families with different parenting styles, or for children with comorbid ADHD or emotional problems or of different ages. | Markov model | No recommendation but does indicate a WTP of £109 per point improvement on the ECBI-I is 50%. This increases to 99% at a WTP of £145. Threshold analysis. | No | Yes | CEAC; DSA | Compared to no intervention, a WTP of £109 per point improvement on the ECBI-I is 50% probability of being cost-effective.  In the ‘high-cost’ scenario, the return on investment is substantial, with average net savings of between £5000 and £7000 per child. |
| Griebsch 2007 | Timely diagnosis of life-threatening congenital heart defects | No | No | Decision tree | Pulse oximetry 'appears cost-effective' | No | Yes | CEAC; DSA. Conducted VOI analysis in the form of EVPI and EVPPI. | Compared to clinical examination alone, the ICER for pulse oximetry is £4,894 per additional timely diagnosis; for screening echocardiography it is £4,496,666 per additional timely diagnosis. |
| Grill 2006 | Quality weighted detected child months | No | No | Markov model | Not stated. Threshold analysis. | No | Yes | CE-plane (PSA); CEAC; DSA | Compared to community, the ICER is £2423 per detected child; £25 per quality weighed detected child month |
| Hoddinott 2012 | Any breastfeeding; exclusive breastfeeding | No | Intervention targeted at women living in SIMD 1–3 postcode areas. | N/a | Not stated | No (trial based evaluation) | No | n/a | Compared to reactive only telephone support, the ICER is £87 per additional woman any breastfeeding; £91 per additional woman exclusively breast feeding |
| Hodgson 2020 | QALYs | No | No | Dynamic transmission model | No recommendation. Results presented as the maximum purchasing price per course for programmes to be cost-effective. Threshold analysis. | No | Yes | Equivalent of CEAC with maximum purchase price for vaccine and box plots (sensitivity analysis) | Results presented as the maximum purchasing price per course for programmes to be cost-effective (compared to status quo). For: MAB-VHR-S (£4342.97); MAB-HR-S (£201.15); MAD; MAB-HR-S+ (£87.03); VAC-INF-S (£94.76). |
| Hollingworth 2012 | Life years gained | No | No | Microsimulation model | Yes | No | No | n/a | Compared to no/minimal intervention, the ICER is £66,567 per life year gained (BMI standard deviation score reduction of 0.03); £13,589 per life year gained (0.13 BMI standard deviation score reduction) |
| Jacklin 2007, NICE 2008 | QALYs; premenopausal breast cancer averted; infant infections averted | No | Intervention targeted at the poorest areas of Sheffield | Unclear | Yes | Unclear | No | DSA | No ICER reported. Investment of £20,000 in a peer support scheme of this type produces net societal savings of £5,500. In addition the model suggests that the scheme would avert 0.057 cases of pre-menopausal breast cancer in mothers (2.7 cases per 10,000) and almost 6 cases (285 cases per 10,000) of infections requiring hospitalisation in the first year of life. |
| Jit 2007 | QALYs | No | No | Cohort model | No | No | Yes | CE-plane (PSA); CEAC; DSA | Compared to no vaccination, the ICER is £79,905 per QALY gained; £525 per episode prevented; £3,803 per hospitalisation prevented (vaccination using RotaTeq).  £60,928 per QALY gained; £391 per episode prevented; £3,647 per hospitalisation prevented (vaccination using Rotarix). |
| Jit 2009 | QALYs | No | No | Cohort model | No | No | No | Not presented | Compared to current care, the ICER is EUR110,000 per QALY gained (Rotarix vaccination programme) and EUR160,000 per QALY gained (RotaTeq vaccination programme) |
| Jit 2010 (Update of 2009 paper with new efficacy evidence) | QALYs | No | No | Cohort model | No | No | No | Not presented | Compared to current care, the ICER is EUR110,000 per QALY gained (Rotarix vaccination programme) and EUR150,000 per QALY gained (RotaTeq vaccination programme) |
| Kay 2018 | QALYs | No | The intervention was targeted at those at high risk of oral disease (children in the most deprived quintile in England). | Modelling of relative risk reduction of caries | Range of costs given for the interventions to be deemed cost-effective | No | No | Not presented | Compared to no intervention, spending less than £55 per child on supervised tooth brushing is cost-effective; spending less than £100 on varnish would be cost-effective over 3 years |
| Kendrick 2017 i | QALYs | No | Interventions were aimed at people in social housing and in the most deprived areas | Markov model | Not really stated. Threshold analysis.  Note, intervention e) was deemed not to be cost-effective | No | Yes | CEAC; DSA | a) Compared to usual care, the ICER for education + equipment is £34,200 per QALY gained. Note, this was the only non-dominated intervention.  b) Compared to usual care, the ICER for education is £40,271 per QALY gained. Note, this was the lowest ICER.  c) Compared to usual care, the ICER for education is £284,068 per QALY gained. Note, this was the lowest ICER.  d) Compared to usual care, the ICER for education is £41,330 per QALY gained. Note, this was the lowest ICER.  e) All interventions were more costly and less effective than usual care |
| Kendrick 2017 ii | Probability of having a fire escape plan | No | The children’s centres provide community-based integrated services, information  and support for families with young children. They aim to improve outcomes for young children and their  families, with a particular focus on the most disadvantaged, to reduce inequalities in health. | n/a | IPB only dominates (cost saving and better outcomes) | No | No | CEAC; DSA | Compared to usual care, the ICER for injury prevention briefing only is £1260 per additional fire escape plan; the ICER for injury prevention briefing + is £616.13 per additional fire escape plan |
| Knerer 2012 | QALYs | No | No | Markov model | Yes | No | Yes | DSA | Compared to PCV-13, the pneumococcal conjugate vaccine dominates (positive QALYs, negative costs). |
| Knowles 2005 | Timely diagnosis | No | No | Decision tree | Not stated. Threshold analysis. | No | Yes | CE-plane (PSA); CEAC; DSA. Conducted VOI analysis in the form of EVPI. | Compared to clinical examination, the ICER is £4,894 per timely diagnosis |
| Kowash 2006 | Monetary and decayed, missing or filled tooth or tooth surface | No | The intervention is targeted at women living in a deprived area of Leeds | n/a | Unclear | No | No | n/a | The benefit/cost ratio is 5.6.  Cost-effectiveness ratio is 1.8. |
| Lorgelly 2007 | Gastroenteritis episode avoided; GP visit avoid; hospitalisation visit avoided; life years saved | No | The authors argued that the societal perspective provides important equity information: differing cost-effectiveness across perspectives reflects the fact the rotavirus gastroenteritis is a significant burden on parents and families. | Decision tree | Yes (but they use that to mean cost saving) | No | No | DSA | Compared to no vaccination programme, the ICER is £60.41 per episode avoided; £177,212 per life year saved. |
| McAuley 2004 | Parenting Stress Index; Edinburgh Postnatal Depression Scale; Rosenberg Self-Esteem; Brief Infant–Toddler Social and Emotional Assessment Scale; Maternal Social Support Index | No | The Study families were referred by Home-Start organisers or health visitors. The predominant reason for referral for all the families in the study fell within the five categories: maternal mental/physical health, social isolation, multiple births/young children and a child/children with special needs. A quarter of families were in council housing, 7% in housing association housing, 85 per cent of single-parent families | n/a | No | No | No | n/a | Compared to no home start support, the intervention was assumed to be dominated (no effect difference and increases costs in the Home Start arm) |
| McIntosh 2003 | Life years saved | No | No | Unclear | Unclear | No | No | n/a | Compared to no vaccination, the ICER is £31,512 per life year saved |
| Melegaro 2004 | Life years gained; QALYs | No | No | Cohort model | Not likely to be deemed cost-effective from the NHS perspective | No | Yes | CEAC; DSA | Compared to no vaccination, the ICER is £70,699 per life year gained; £31,021 per QALY gained.  Other scenarios presented all with higher ICERs. |
| Morell 2000a & Morell 2000b | SF-36; Duke functional social support; Edinburgh postnatal depression scale; number breastfeeding only; number formula milk feeding only | No | No | n/a | Not stated | No | No | n/a | No evidence of differences in health status scores (SF-36, Edinburgh postnatal depression scale, and Duke functional social support scale) and rates of breast feeding between the two groups. The difference in total NHS costs between the groups was £178.61. |
| Mujica 2006 | Proportion of mothers in paid employment or education at 18 months | No | The centres in the intervention are established in areas of high levels of deprivation | n/a | The societal costing was estimated to be cost saving. The public sector evaluation didn't report as there was no specified WTP. Threshold analysis. | No | Yes | CEAC; DSA | Compared to childcare secured by the participants themselves, the ICER was £38,550 per additional woman in paid  Societal perspective shows it to be cost saving. |
| O'Neill 2017 | Proportion caries free; number of carious surfaces; number of episodes of pain | No | No | n/a | Only for carious surfaces at £1,000 per carious surface avoided. Only carious surfaces considered as this was the only statistically significant result. Threshold analysis. | No | Yes | CE-plane (PSA); CEAC; DSA | Compared to advice only, the ICER is £2,092.59 per caries free person; £250.58 per carious surface; £259.07 per number of pain episodes |
| Pandor 2004 + Pandoor 2006 | Life years gained; cases of inborn error of metabolism detected | No | No | Unclear | Probably cost-effective when used for phenylketonuria (PKU)  and medium-chain acyl-coenzyme A dehydrogenase (MCAD)  but not likely for PKU alone. Not likely with the addition of other metabolic diseases | No | Yes | CE-plane (PSA); CEAC. Conducted VOI analysis in the form of EVPI. | Compared to screening for PKU only, the ICER for PKU+MCAD is –£7,359  per case of inborn error of metabolism detected; ICER for cost per life year gained are not reported. |
| Phillips 2011 | Risk reduction (scalds) | No | This intervention is for families with children under 5 years of age living in accommodation provided by the Glasgow Housing Association. | n/a | Cost saving | No | No | n/a | Compared to a waiting list, the scald prevention intervention results in net savings of £7273 per scald avoided (NHS perspective);  £53 949 per scald avoided (lifetime perspective).  The net benefit (cost) per £1 spent is £1.41 for an NHS perspective and (£0.47) for a lifetime perspective. |
| Pitman 2013 | QALYs | No | No | Dynamic transmission model | Not stated.  TIV dominated; LAIV cost saving | No | Yes | CEAC; DSA | Compared to current policy, TIV in 2-4 year olds is dominated. Compared to current policy, LAIV in 2-4 year olds is cost saving (with positive QALYs). |
| Pokhrel 2015 | Cost savings. This includes a cost derived using NMB (assuming 20,000/QALYs) for the breast cancer benefits). | No | Infants of parents from low-income backgrounds, who are young, white, with fewer educational qualifications and who were themselves formula fed, are least likely to be breastfed. | Markov model | Yes | Unsure | No | DSA | Report outcomes using 3 different types of policies,: policy A, B and C (impacts on actue diseases (GI, LRTI and AOM)); Policy D (impacts NEC) and Policy E (impacts BC). Results not combined.  Policy A2 saves £11.04m; policy D2 saves £6.12m and policy E2 saves £31.42m (this includes QALYs gained) |
| Renwick 2018 | Average 16–24 h levels of particulate matter of < 2.5 μm diameter (PM_2.5_)  ; the number of quitters | No | Intervention targeted at deprived communities in Nottingham City and County in England. Caregivers aged 18 and over, with a child aged under five living in their household, reported smoking tobacco inside their home and were not willing to quit | n/a | Not stated | No | Yes | CE-plane (PSA); DSA | Compared to usual care, the ICER is £131 per additional 10μg/m3 reduction of 16-24 h PM2.5; £71 per additional quitter |
| Roberts 2012 | Case of timely diagnosis | No | No | Decision tree | Yes | No | Yes | CEAC; DSA | Compared to clinical examination alone, the ICER is £24,900 per timely diagnosis of significant congenital heart defects |
| Saramago 2014 | QALYs | No | Social inequalities exist in the possession of functioning smoke alarms in families with children under 5 in the UK | Decision tree | Not stated | No | Yes | CEAC; DSA | Compared to usual care, the only non-dominated interventions are education plus low cost/free safety equipment with an ICER of £34,200 per QALY gained; Education plus low cost/free safety equipment plus fitting plus home inspection has an ICER of £3,466,635 per QALY gained. |
| Siddiqui 2011 | QALYs | No | No | Markov model | No | No | No | DSA | Compared to current vaccination practice, the ICER is £263,000 per QALY gained (for universal infant vaccination programme); £90,000 per QALY gained (for the selective infant programme) |
| Simkiss 2013 | QALYs | No | The intervention was implemented in early years centres in four deprived areas of South Wales | n/a | No evidence of value for money | No | Yes | Threshold analysis; DSA | Compared to a waiting list, the ICER is £34,913 over 5 years and  £18,954 over 10 years |
| Simpson 2005 | QALYs | No | No | Decision tree + markov model | Not stated | No | No | DSA | Compared to no screening, the ICER is £6,864 per QALY gained |
| Thomas 2018 | Costs | No | No | n/a | For certain subgroups (BD, EI and PN) | No | No | DSA | Compared to no vaccination procedure, the benefit/cost ratio is: 7.726 for bronchopulmonary dysplasia; 0.694 for congenital heart disease; 1.391 for extreme immaturity; 1.426 for premature babies; 0.465 for all other RSV admissions. All results for year of 2012/2013. |
| Tickle 2016 | Caries-free person; carious surfaces; episodes of pain | No | The study did look at the effects of the intervention across IMD quintile groups but didn't consider these groups in the economic evaluation, and the discussion included a discussion of the uptake and effect of a universal intervention across SE groups. | n/a | No (not for the outcomes of caries avoided) | No | Yes | CEAC; DSA | Compared to prevention advice alone, the ICER is £2092.59 per proportion caries free; £250.58 per number of carious surfaces; £259.07 per episode of pain |
| Trotter 2002 | Life years saved | No | No | Cohort model | Modelling of the cost effectiveness of the campaign supports the introduction of the vaccine | No | No | DSA | Compared to no vaccination, the ICER is £14,630 per life year saved for 0-4 month programme, £9,493 per life year saved for the 5-11 month programme, £5,826 per life year saved for the 1-4 year programme. |
| Trotter 2006a | Life years gained and QALYs | No | No | Dynamic transmission model | Not stated | The paper was exploring the differences in modelling with dynamic model compared to previous paper with static model. But not difference in model structure in the paper | No | DSA | Compared to no vaccination, the ICER for the 2, 3, 4 months programme is £38,164 per life year saved; £31,152 per QALY gained. |
| Trotter 2006b | Life years gained | No | No | Dynamic transmission model | Not stated | Two models were explored. The base case and then an additional model to consider the assumptions around the waning of duration of protection against carriage acquisition | No | Not presented | Compared to the current schedule, the ICER for Strategy 2 is £4,498,000 per life year gained; Strategy 3a (2,4,13 months) -£ 2,000 per life year gained; Strategy 3ab (3, 13 months) -£4,811,000 per life year gained; Strategy 4 -£16,419,000 per life year gained |
| Tudor Edwards 2016 | Strengths and Difficulties Questionnaire (SDQ) and Eyberg Child Behaviour Inventory (ECBI), and the Arnold-O’Leary Parenting Scale (APS). | No | No | n/a | Not stated. Threshold analysis. | No | Yes | CE-plane (PSA); CEAC; DSA | Compared to waiting list, the ICER is £1,295 per one point improvement in SDQ; £237 per one point improvement in ECBI-I; £9,477 per one point improvement in APS |
| Uus 2006 | Cases detected | No | No | n/a | Results compared favourably' - not explicitly stated | No | No | n/a | Compared to infant Distraction Test Screening, the ICER is £12,527 per case detected |
|  |  |  |  |  |  |  |  |  |  |
